# CD4 T cell autophagy is integral to memory maintenance

**DOI:** 10.1038/s41598-018-23993-0

**Published:** 2018-04-13

**Authors:** Diane Murera, Florent Arbogast, Johan Arnold, Delphine Bouis, Sylviane Muller, Frédéric Gros

**Affiliations:** 1 CNRS, Immunopathology and therapeutic chemistry/Laboratory of excellence MEDALIS, Institute of molecular and cellular biology (IBMC), Strasbourg, France; 2 University of Strasbourg, Strasbourg, France; 3 CNRS-University of Strasbourg, Biotechnology and cell signaling, Illkirch, France/Laboratory of excellence MEDALIS, Institut de science et d’ingénierie supramoléculaire, 67000 Strasbourg, France; 4 University of Strasbourg Institute for Advanced Study (USIAS), Strasbourg, France

## Abstract

Studies of mice deficient for autophagy in T cells since thymic development, concluded that autophagy is integral to mature T cell homeostasis. Basal survival and functional impairments *in vivo*, limited the use of these models to delineate the role of autophagy during the immune response. We generated *Atg5*^*f/f*^ distal Lck (dLck)-cre mice, with deletion of autophagy only at a mature stage. In this model, autophagy deficiency impacts CD8^+^ T cell survival but has no influence on CD4^+^ T cell number and short-term activation. Moreover, autophagy in T cells is dispensable during early humoral response but critical for long-term antibody production. Autophagy in CD4^+^ T cells is required to transfer humoral memory as shown by injection of antigen-experienced cells in naive mice. We also observed a selection of autophagy-competent cells in the CD4^+^ T cell memory compartment. We performed *in vitro* differentiation of memory CD4^+^ T cells, to better characterize autophagy-deficient memory cells. We identified mitochondrial and lipid load defects in differentiated memory CD4^+^ T cells, together with a compromised survival, without any collapse of energy production. We then propose that memory CD4^+^ T cells rely on autophagy for their survival to regulate toxic effects of mitochondrial activity and lipid overload.

## Introduction

Autophagy is a catabolic process, required to produce energy notably under nutrient deprivation. Moreover, basal autophagy is important to remove protein aggregates, damaged organelles such as defective mitochondria or excess of endoplasmic reticulum (ER), in processes called mitophagy and reticulophagy, respectively. Autophagy is also involved in the regulation of lipid stores through the digestion of lipid droplets via the so- called “lipophagy”^1^. Basal autophagy has been shown to be crucial in long-lived cells, such as neurons, or metabolically active cells, such as hepatocytes. Immune cells like T lymphocytes exhibit differential energy demands according to their developmental stage or their activation status. Thus, naive T cells require glycolysis early after activation, to quickly sustain the energetic demand while, in contrast, memory T cell clones, use differential energy production systems to survive for months or years after priming^2^. Memory T cells are particularly dependent on fatty acid oxidation (FAO) that takes place in mitochondria, to generate adenosine tri-phosphate (ATP). Moreover, removal of damaged cellular components may also require autophagy at long-term.

Autophagy has been initially shown to play a role in peripheral T cell homeostasis in mouse chimera models^3^. By the use of several conditional deletion models, it was thus concluded that autophagy is essential for both CD4^+^ and CD8^+^ T cell survival and proper function^4–10^. However, these models relied on promoters driving autophagy-related genes (*Atg*) deletion early during T cell differentiation. These models alone were thus not sufficient to distinguish if functional or survival defects were due to early developmental issues, or consequent to peripheral homeostatic disturbance. One study addressed this issue by deleting, with Estrogen Receptor-cre promoter, the essential autophagy gene *Atg3*, therefore only in mature T cells^7^. This study led to new findings about the requirement of autophagy for ER homeostasis, several days after initial activation. However, these experiments were conducted *in vitro* and therefore integrated immune responses could not be studied. More recently, three other studies addressed this question *in vivo* for CD8^+^ T cells, by transfer experiments and using conditional deletion models only active at the CD8 T cell effector stage. They concluded that CD8^+^ T cells require autophagy for their survival as memory cells^11–13^. These observations constitute an interesting parallel to other long-lived cell types, like neurons, in which autophagy is particularly required. Although investigated in CD8^+^ T cells, the role for autophagy in the memory of the CD4^+^ T cell compartment is not known yet.

In this work, we generated mice with a deletion of *Atg5*, only in mature T cells, using the distal *Lck* (dLck) promoter conditional knock-out strategy^14^. With this new model, we wanted to precisely define the role of autophagy in peripheral T cell homeostasis and function, in the absence of any developmental issue. We particularly focused our attention on the essential role of autophagy in memory CD4^+^ T cell survival. In addition to the proven role of autophagy in CD8 memory maintenance, we describe here a role for this essential survival process in humoral immunity, through the promotion of long-term memory CD4^+^ T cell survival. We show that in memory T cells *ex vivo*, as well as in cells obtained by *in vitro* differentiation systems, autophagy insures the control of lipid load and of a functional mitochondrial pool. These observations endow autophagy with a central role in the survival of memory CD4^+^ T cells.

## Results

### Autophagy is not required for peripheral CD4^+^ T cell homeostasis

**Figure 1:**
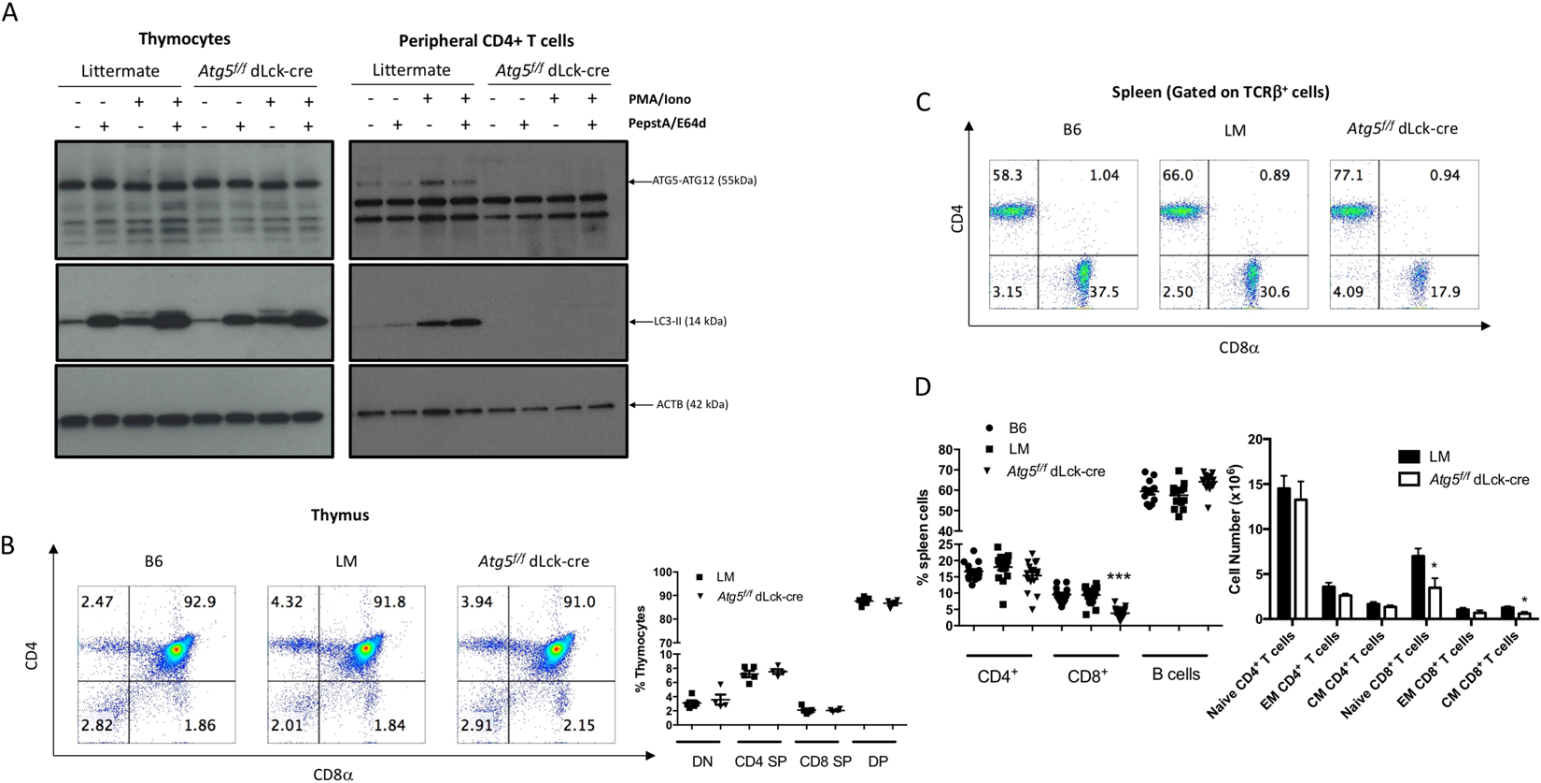
(**A**) Thymocytes or peripheral CD4^+^ T cells were isolated from spleens of littermate or *Atg5*^*f/f*^ dLck-cre mice. Cells were stimulated when indicated by 50 ng/mL PMA and 1 µg/mL Ionomycin for 18 hours. During the last 4 hours of stimulation, cells were treated when indicated with pepstatin A and E64d. Cell lysates were then processed by SDS-PAGE and blotted against ATG5 and LC3. Representative experiment of at least three replicates. Complete images of different blotting of the same membrane, including data with wild type C57BL/6 (B6) mice, are shown in Figure S1. (**B**) Left: Representative dot plots of thymocytes stained by anti-CD4 and anti-CD8 Abs (left) from B6, littermate (LM), *Atg5*^*f/f*^ dLck-cre mice. Right: proportions among thymocytes of double negative (DN), CD4 single positive (CD4 SP), CD8 single positive (CD8 SP) or double positive (DP) cells (n = 5 LM and n = 4 *Atg5*^*f/f*^ dLck-cre mice) (**C**). Representative dot plots of spleen cell staining by anti-CD4 and anti-CD8 Abs, after gating on TCRβ^+^B220^−^ cells from B6, littermate LM, *Atg5*^*f/f*^ dLck cre mice. (**D**) Percentages of CD4^+^, CD8^+^ T cells, and B cells among spleen cells (n = 13 B6, n = 17 LM and n = 17 *Atg5*^*f/f*^ dLck-cre mice). (**E**) Absolute numbers of naive, effector memory (EM, CD44^hi^ CD62^lo^) and central memory (CM, CD44^hi^CD62L^hi^) CD4^+^ and CD8^+^ T cells in spleens (n = 12 for each genotype) among TCR-β^+^-gated cells. Each point represents the value obtained with one mouse. LM and *Atg5*^*f/f*^ dLck-cre mice are compared by Mann Whitney U Test. *p < 0.05, ***p < 0.001.

To resolve the question whether autophagy is required for mature T cell homeostasis, we crossed *Atg5*^*f/f*^ animals with mice harbouring a transgene allowing CRE expression, under the control of the distal part of the Lck promoter (dLck-cre), only active in mature T cells. We first assessed the efficiency of the deletion. As shown in Figs 1A and S1, no ATG5-ATG12 conjugate was detected by immunoblot in peripheral CD4^+^ T cells isolated from *Atg5*^*f/f*^ dLck-cre mice, contrary to littermates. No conversion from LC3-I to LC3-II (Light Chain 3 abbreviated from microtubule-associated protein light chain 3) was detectable, even after phorbol-12-myristate-13-acetate (PMA)/Ionomycine activation and/or under protease inhibitor treatment, confirming the efficiency of autophagy inactivation in T cells. In thymocytes, no difference was seen between *Atg5*^*f/f*^ dLck-cre mice, and littermate mice, in ATG5-ATG12 or in LC3-II levels. This confirms the expected specific ATG5 deletion only at the mature stage of T cells. We then investigated the impact of this deletion during T cell development. In accordance with the normal expression of ATG5 in the thymus, we did not observe any difference in thymic cellularity (not shown), or in the proportions of each major developmental subpopulation (Fig. 1B). This suggests a normal development of T cells in our model. We then assessed the proportions of lymphocyte populations in secondary lymphoid organs. We observed a decrease in the proportion and number of spleen CD8^+^ T cells (Fig. 1C,D) from *Atg5*^*f/f*^ dLck-cre mice compared to controls. However, in sharp contrast to all conditional deletions reported so far (with cre expressed under the control of *cd4* or *pLck* promoters), we found no difference in CD4^+^ T cell number and proportion among other populations. This finding was extended to another lymphoid organ, namely lymph node (Figure S2). Interestingly, both CD8^+^ naive and memory-like (CD44^hi^) T cell numbers were decreased in *Atg5*^*f/f*^ dLck-cre mice (Fig. 1E). This phenotype suggests a global role for autophagy in the homeostasis of CD8^+^ T cell populations that is not observed in CD4^+^ T cells.

### Autophagy is dispensable for early CD4^+^ T cell activation

**Figure 2:**
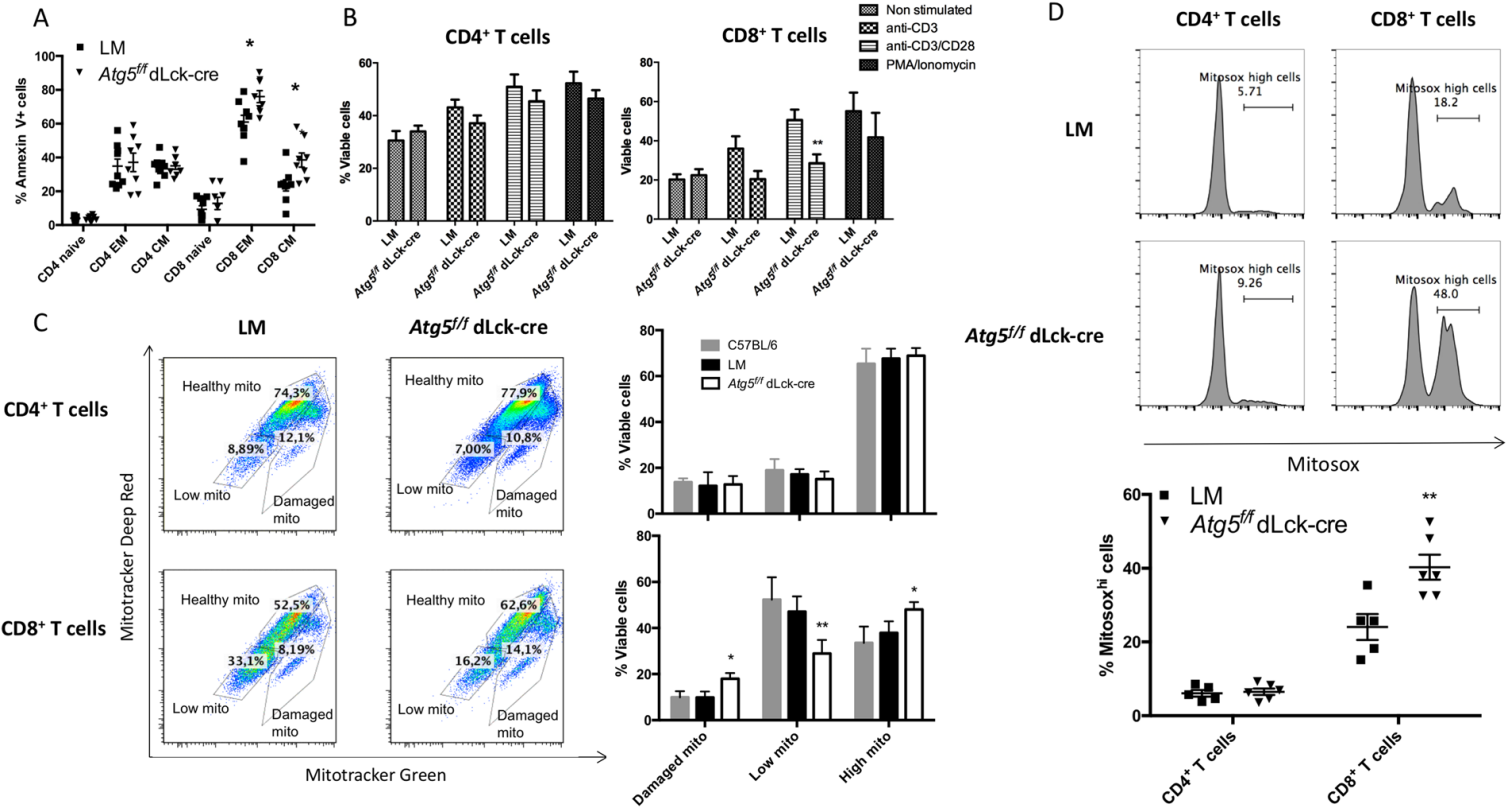
(**A**) Percentages of dead cells measured by Annexin V staining *ex vivo* from the spleen for the following populations: effector memory (EM, CD44^hi^ CD62^lo^) and central memory (CM, CD44^hi^CD62L^hi^) after gating on TCRβ^+^CD4^+^ cells or TCRβ^+^CD8^+^ cells, from littermate (LM) and *Atg5*^*f/f*^ dLck-cre mice (n = 8 for each gentotype). (**B**) Percentages of viable cells defined as Annexin V^−^/propidium iodide (PI)^−^, defined after stimulation of purified CD4^+^ (left) or CD8^+^ T cells (right) under the indicated conditions (non-stimulated, stimulated by anti-CD3, by anti-CD3 and anti-CD28, or by 50 ng/mL PMA and 1 µM ionomycin, n = 4 for each genotype). (**C**) Staining of CD4^+^ or CD8^+^ T cells purified from LM or *Atg5*^*f/f*^ dLck-cre mice by mitotracker deep red and mitotracker green. Gates could be defined for cells with either high load of mitochondria (mitotracker deep red^hi^, mitotracker green^hi^), low mitochondrial content (mitotracker deep red^low^, mitotracker green^low^), or with damaged mitochondria (mitotracker green^hi^, mitotracker deep red^low^). On the right, histograms show the results of independent experiments (n = 4 for each genotype). (**D**) *Ex vivo* staining of spleen cells from LM or *Atg5*^*f/f*^ dLck-cre mice, by Mitosox, after gating on TCRβ^+^CD4^+^ cells or TCRβ^+^CD8^+^. (Top) representative histograms are shown and (bottom) a summary of different experiments is shown. LM and *Atg5*^*f/f*^ dLck-cre mice are represented and compared by Mann Whitney U Test. **p < 0.01, *p < 0.05.

**Figure 3:**
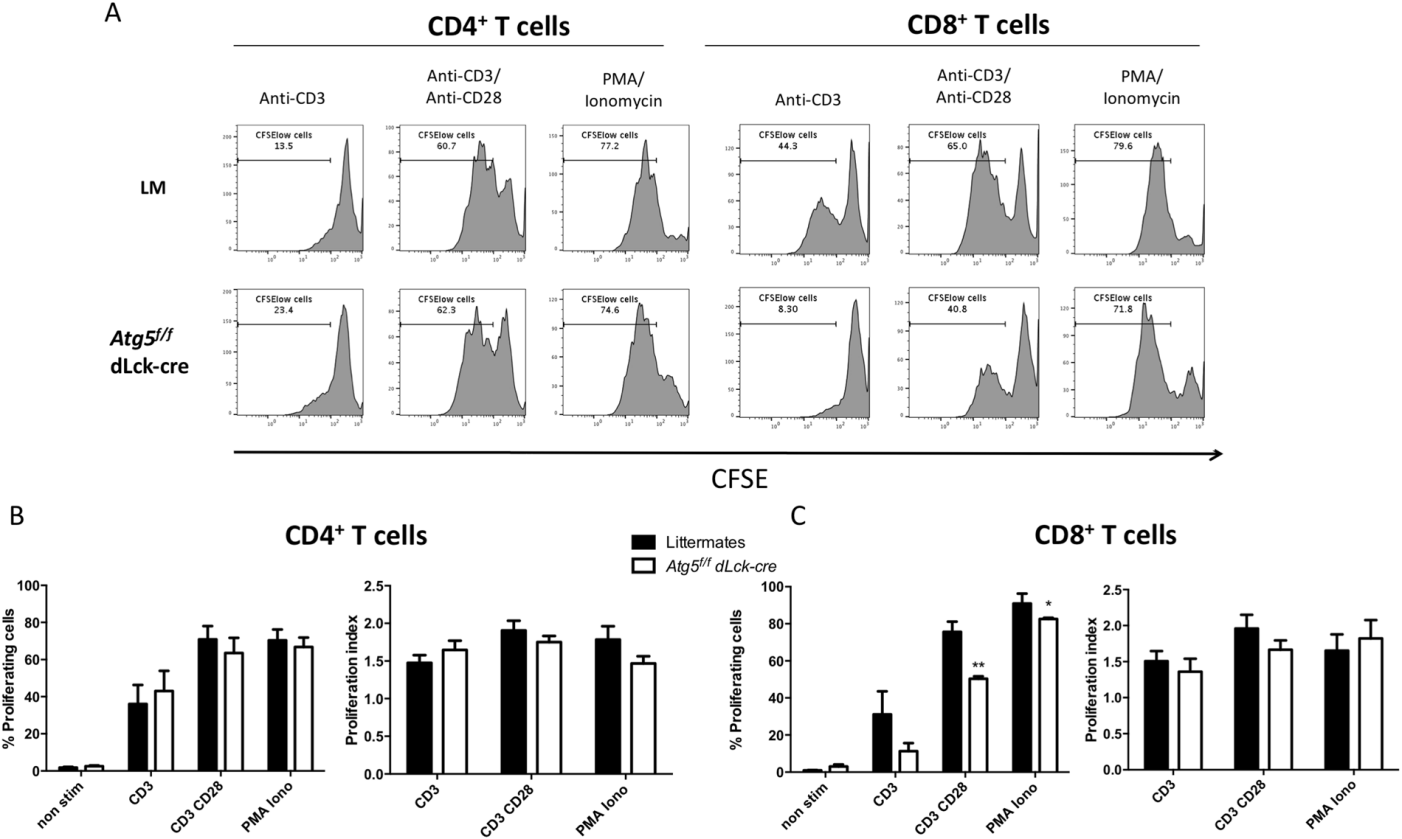
(**A**) T cell proliferation monitored by carboxyfluoescein succinimidyl ester (CFSE) staining after stimulation by anti-CD3 antibody, a combination of anti-CD3 and anti-CD28 antibodies or 50 ng/mL PMA and 1 µg/mL ionomycin, for 72 hours. Percentages shown indicate CSFE^low^ cells, i.e. cells that have proliferated. (**B**,**C**) Results obtained on several experiments are synthesized in histograms (n = 5 for each genotype). Littermate (LM) and *Atg5*^*f/f*^ dLck-cre mice are represented and compared by Mann Whitney U Test. **p < 0.01, *p < 0.05. Percentages of proliferating cells are shown (**B**) as well as proliferation indexes (**C**), representing the total number of cell divisions, divided by the number of cells that have proliferated.

We then studied the possible causes of CD8^+^ T cell number decrease in *Atg5*^*f/f*^ dLck-cre mice. Compared to littermate control mice, a higher number of memory-like CD8^+^ T cells (effector memory (EM) and central memory (CM)) displayed an apoptotic phenotype in *Atg5*^*f/f*^ dLck-cre mice (Fig. 2A). Less naive CD8^+^ T cells were found in the absence of autophagy, suggesting that cells first acquired an activated phenotype before entering apoptosis, progressively leading to the depletion of the naive cell pool. These data are reminiscent of the ones obtained by Puleston *et al*.^11^ concluding that CD8^+^ T cell deficiency in the absence of ATG5 is linked to an activated phenotype. In contrast, no impact of autophagy deletion was seen on CD4^+^ T cell apoptosis, leading to the conclusion that ATG5 could be essential for homeostatic survival of CD8^+^ T cells, but not for CD4^+^ T cells. In addition, the survival difference observed between CD4^+^ and CD8^+^ T cells is not due to an escape to cre-mediated deletion in purified CD4 T cells *in vivo*, as *Atg5* transcript levels are equally low in both T cell populations at steady state (Figure S3). We then isolated CD4^+^ and CD8^+^ T cells from *Atg5*^*f/f*^ dLck-cre and control littermate mice and activated them with TCR-related stimulation conditions. We did not detect any survival defect in CD4^+^ T cells (Fig. 2B). However, CD8^+^ T cells exhibited a statistically significant decrease of viability after TCR stimulation in combination with co-stimulation. Thus, autophagy deficiency affects mainly CD8^+^ T cells, at homeostatic conditions, and under stimulation. As survival defects could be linked to mitochondria function, we then assessed the basal mitochondrial load in isolated T cells. We used a double staining with mitotracker (MT) deep red, sensitive to mitochondrial membrane potential, together with MT green, staining mitochondria irrespectively of the membrane potential and allowing to normalize the measurement of membrane potential to the mitochondrial load. First, we found that CD4^+^ T cells exhibit a higher mitochondrial mass in control T cells, in comparison to CD8^+^ T cells (“High mito” population, Fig. 2C). Interestingly, we detected more T cells with a high mitochondrial load, or T cells containing mitochondria with decreased membrane potential (damaged mito), in CD8^+^ T cells isolated from *Atg5*^*f/f*^ dLck-cre mice compared to control mice. This indicates that ATG5 is required after thymus egress, in CD8^+^ T cells and not in CD4^+^ T cells, to reduce mitochondrial mass. We also found increased mitochondrial reactive oxygen species (ROS) production by Mitosox staining in spleen CD8^+^ T cells in the absence of ATG5, while no difference was observed in CD4^+^ T cells (Fig. 2D). Altogether, these findings could be related to the homeostatic defects observed in CD8^+^ T cells deficient for ATG5 at the mature stage. In addition to survival defects, we noticed a specific proliferation impairment *in vitro* after three days of TCR-related stimulations in CD8^+^ T cells, but not in CD4^+^ T cells (Fig. 3A,B), in terms of number of cells that enter into a at least one cycle of division. However, as no difference is seen on proliferation index in either CD4^+^ or CD8^+^ T cells, we can speculate that the proliferation defect is linked to the global survival decrease of some CD8^+^ T cells, and not to an abnormal intrinsic proliferative capacity. Moreover, the normal proliferative ability of autophagy-deficient CD4^+^ T cells is not due to an enrichment of ATG5 expressing cells, as deletion is equivalent before and after stimulation (Figure S4). Taken together, these results endow autophagy with a role for CD8^+^ T cell homeostasis. Autophagy is in contrast dispensable for short-term survival and activation of CD4^+^ T cells.

### CD4^+^ T cell autophagy increases the efficiency of long-term humoral responses

**Figure 4:**
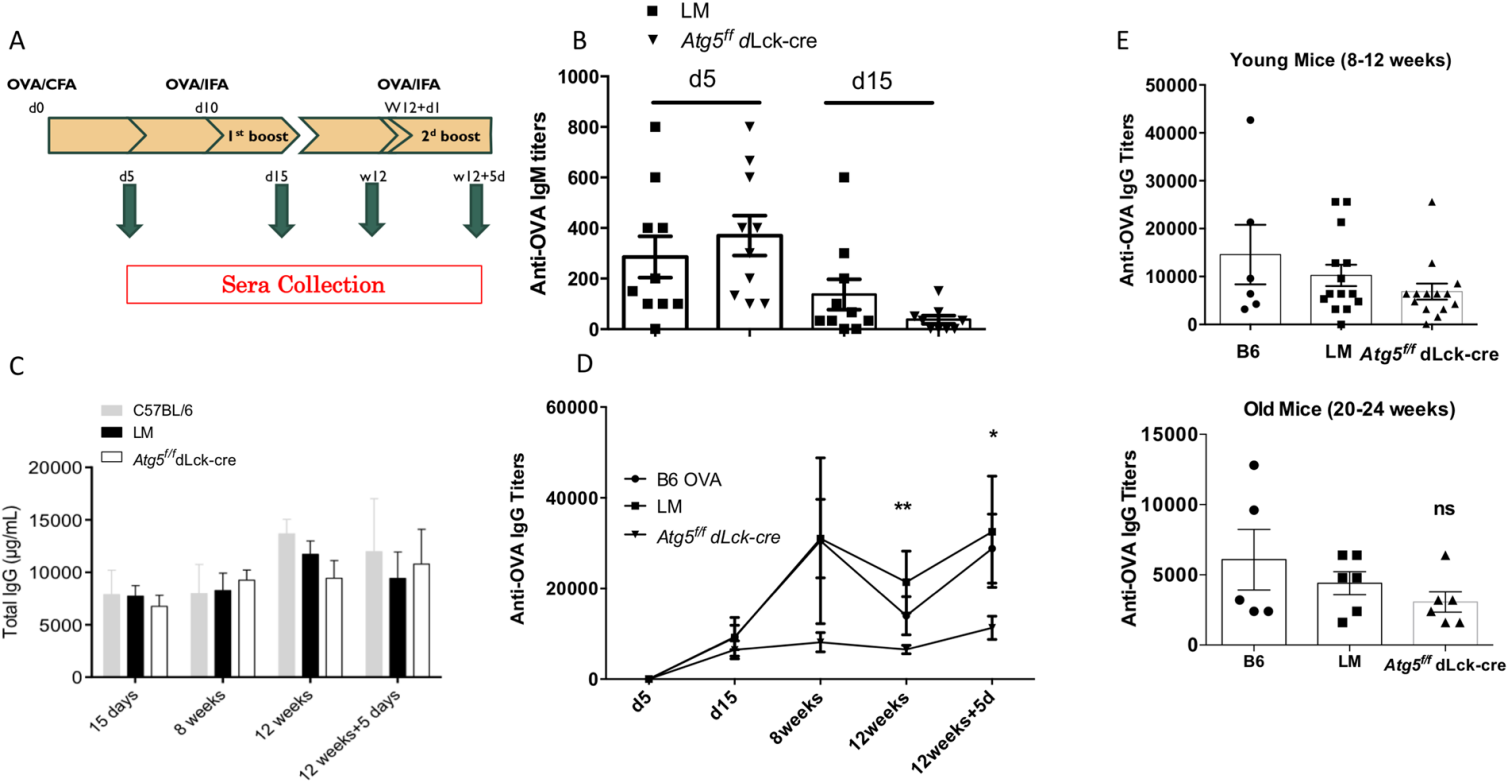
(**A**) Immunization protocol used for the results presented in B and C, to show the impact of T cell autophagy on long term humoral response. Mice received OVA in complete Freund’s adjuvant (CFA) i.p. at day 0, and at day 10 OVA in incomplete Freund’s adjuvant (IFA). Mice were then injected i.p. at week 12 + 1 day with OVA in IFA. Sera were collected at days 5 and 15 and then at week 12 and week 12 + 5 days. (**B**) Anti-OVA IgM titers as measured by ELISA at days 5 and 15 (n = 10 littermates (LM) and *Atg5*^*f/f*^ dLck-cre mice). (**C**) Total IgG levels measured by ELISA at different time points after the injection (n = 2 B6, n = 6 LM and n = 6 *Atg5*^*f/f*^ dLck-cre mice). (**D**) Longitudinal monitoring of anti-OVA IgG titers as measured by ELISA (n = 6 B6, n = 12 LM and n = 14 *Atg5*^*f/f*^ dLck-cre mice). LM and *Atg5*^*f/f*^ dLck-cre mice are compared by Mann Whitney U Test. **p < 0.01, *p < 0.05. (**E**) Histograms showing results of anti-OVA IgG, as measured by ELISA obtained after one injection at day 0 and one boost at day 10. Each point corresponds to a measurement for one mouse. Ab titers were measured at day 15. (Top) young mice (8–12-week-old, n = 6 B6, n = 12 LM and n = 14 *Atg5*^*f/f*^ dLck-cre mice) and (bottom) old mice (20 to 24-week-old, n = 5 B6, N = 6 LM and n = 6 *Atg5*^*f/f*^ dLck-cre mice) from different genotypes were compared. ns: non-significant.

**Figure 5:**
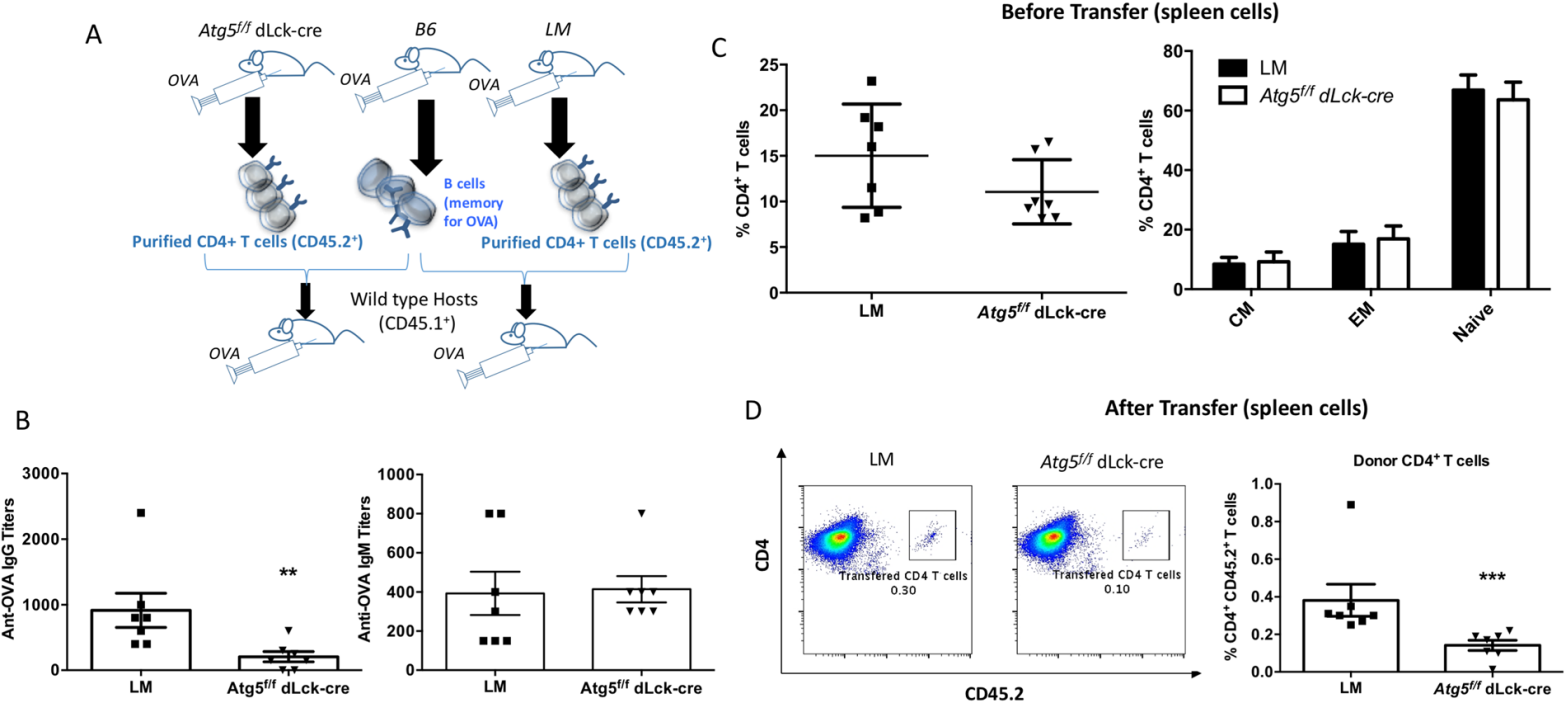
(**A**) Transfer experiment protocol used to determine the intrinsic defect in CD4^+^ T cell memory in the absence of autophagy. C57BL/6 (B6), littermate (LM) or *Atg5*^*f/f*^ dLck-cre mice received OVA in CFA. B cells were isolated from wild B6 mice and CD4^+^ T cells isolated from LM or *Atg5*^*f/f*^ dLck-cre mice. B cells and CD4^+^ T cells were transferred into naive wild type hosts, that were then immunized by OVA in IFA. (**B**) Measurement by ELISA of anti-OVA IgG and IgM in serum, 5 days after immunization of transferred naive hosts with OVA. Each point represents an individual measurement, histograms stand for means and bars represent standard deviation (n = 6 transfers with LM T cells and n = 7 with *Atg5*^*f/f*^ dLck-cre T cells). (**C**) Left: analysis of the proportions of TCRβ^+^CD4^+^ cells in the spleen of donor animals before transfer. Each point represents a single measurement. On the right, activation status of purified CD4^+^ T cells before the injection to host animals. Proportion of naive cells (CD44^lo^CD62L^hi^) central memory cells (CM, CD44^hi^CD62L^hi^) and effector memory   cells (EM, CD44^hi^CD62L^lo^) are indicated. (**D**) Left: Dot plots representing the proportion of donor CD4 T cells (CD45.2^+^ cells among CD4+ cells) at the end of the experiment. Right: summary of the obtained results for the mouse studied. LM and *Atg5*^*f/f*^ dLck-cre mice are compared by Mann Whitney U Test. **p < 0.01, ***p < 0.001.

In our model, neither basal survival of CD4^+^ T cells nor their short-term *in vitro* activation are compromised by the absence of autophagy. This allowed us to define *in vivo* the role of autophagy in T cell help during humoral responses. We immunized mice with the T-dependent antigen ovalbumin (OVA) and performed a boost at day 10 as indicated in Fig. 4A. We monitored the immune response by sampling blood at several time points after OVA administration, and 12 weeks after the start of the experiment. We did not observe any difference at days 5 and 15 in anti-OVA IgM production after immunization, suggesting that there is no major defect in Ig production (Fig. 4B). Absence of any major immunodeficiency or defects in class switching is supported further by the comparable titers of anti-OVA IgG between control mice and mice deficient for autophagy in T cells measured at early time points (Fig. 4C). However, from 8 to 12 weeks, we noticed a significantly reduced anti-OVA IgG titer in mice with *Atg5* deficiency in T cells (Fig. 4D). We then performed a second boost to elicit immune memory and we observed that the response was weaker in *Atg5*^*f/f*^ dLck-cre mice. These results are in accordance with the tendency of a decrease in proportions of germinal center (GC) B cells and the significant lower splenic plasma cell percentage at week 8, when anti-OVA IgG levels start to decline (Figure S5). However, no decrease of TfH cell counts was found in mice with T cells deficient for autophagy, ruling out a global defect on this cell subtype. This decrease of long-term humoral response in the absence of autophagy in CD4^+^ T cells might then be related to a decline in antigen-experienced memory TfH cells (and not the whole population), or other helper cell subsets. We then wondered if the long-term effect of autophagy deletion on specific antibodies could result from a global defect in humoral response in these mice, or to an early T cell senescence in the absence of autophagy in mature T cells. As mentioned before, we did not detect any significant decrease of total IgG levels in *Atg5*^*f/f*^ dLck-cre mice compared to controls, arguing against a general defect in Ig secretion (Fig. 4C). We then compared immunizations of young and older mice (20–24 weeks old) to assess if old mice exhibited a decreased response to first challenges (with only 1 boost at day 10). We did not observe any significant differences between young control mice and mice with autophagy-deficient T cells (Fig. 4E), in accordance with our initial results at day 15 (Fig. 4D). At short-term, we did not see either any differences in old mice when we compared their production of anti-OVA IgG, with or without ATG5 expression by T cells. These results show that *Atg5* deficiency in CD4^+^ T cells does not induce their early senescence. They rather suggest a specific effect on the onset of a memory immune response. We then tested the possibility that an intrinsic defect in CD4^+^ T cell memory is the cause of the long-term humoral response decrease. To do so, we transferred both antigen-experienced CD4^+^ T cells (from B6 or littermate controls, or *Atg5*^*f/f*^ dLck-cre mice), and B cells in naive mice (Fig. 5A). We then immunized the transferred mice with OVA. We observed a detectable anti-OVA IgG response in mice transferred with littermate antigen-experienced CD4^+^ T cells (Fig. 5B), while no signal was detected with mice that received naive B6 T cells (not shown). This result reflects the effective transfer of memory, as IgG are reminiscent of a secondary immune response. Interestingly, the anti-OVA IgG response was weaker when mice were transferred with autophagy-deficient CD4^+^ T cells before immunization. In contrast, we observed a normal anti-OVA IgM response (Fig. 5B). These differences in IgG production were not due to initial deficiency of the CD4^+^ T cell compartment or in T cell activation. Indeed, naive and memory T cell subsets in immunized donor mice, that reflects the activation status of transferred cells (Fig. 5C), were comparable between control and autophagy-deficient animals. In contrast, at the end of the experiment, the proportion of CD4^+^ T cells from the donor was reproducibly lower among CD4^+^ T cells when autophagy-deficient T cells were transferred (Fig. 5D). This suggests that autophagy is needed for the maintenance of injected cells in host animals, and thus for the transfer of memory. Taken together, these results show that an intrinsic defect in autophagy-deficient CD4^+^ T cell compartment impairs the generation of a memory humoral response.

### Autophagy is essential for CD4^+^ T cell central memory survival

**Figure 6:**
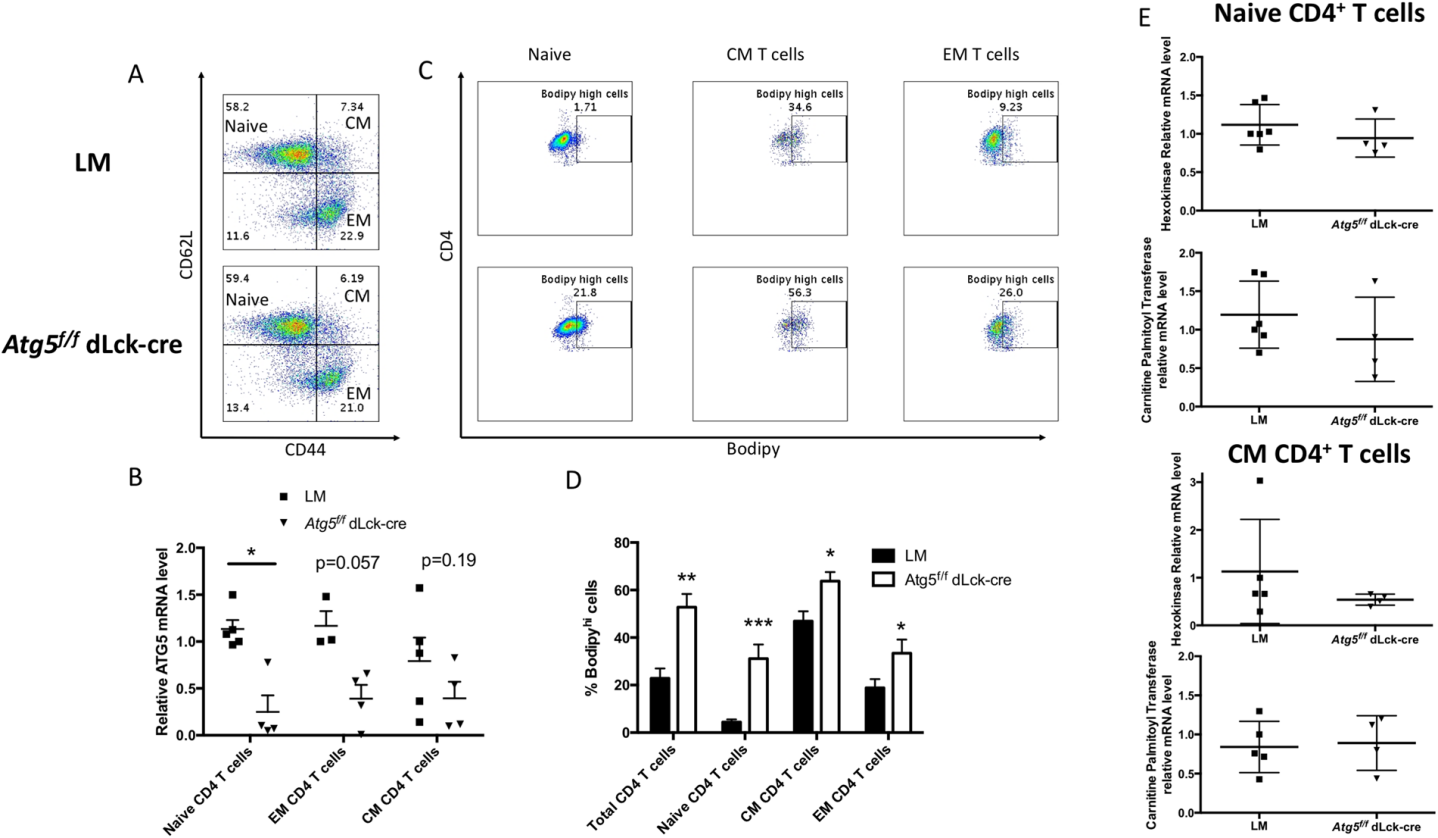
(**A**) Spleen cells from Littermate (LM) and *Atg5*^*f/f*^ dLck-cre mice were stained by anti-TCRβ, anti-CD4, anti-CD62L and anti-CD44 antibodies. Naive (CD44^lo^CD72L^hi^), effector memory (EM, CD44^hi^CD62L^hi^), central memory (CM, CD44^hi^, CD62L^hi^) CD4^+^ T cells, were quantified, and FACS-sorted. (**B**) *Atg5* transcripts were quantified by real-time PCR, after cDNA generation, on the indicated FACS-sorted cell populations. (**C,D**) Spleen cells were stained *ex vivo* by anti-TCRβ, anti-CD4, anti-CD62L and anti-CD44 antibodies and Bodipy. Gating strategy to define percentages of cells with a high staining of bodipy is depicted in **C**, while a summary of the 4 mice tested for each genotype is indicated in **D**. (**E**) *Hexokinase* and *Carnitine palmitoyltransferase* transcripts, were quantified by real-time PCR, after cDNA generation, on the indicated cell populations sorted by FACS. LM and *Atg5*^*f/f*^ dLck-cre mice are compared by Mann Whitney U Test. *p < 0.05, **p < 0.01, ***p < 0.001.

**Figure 7:**
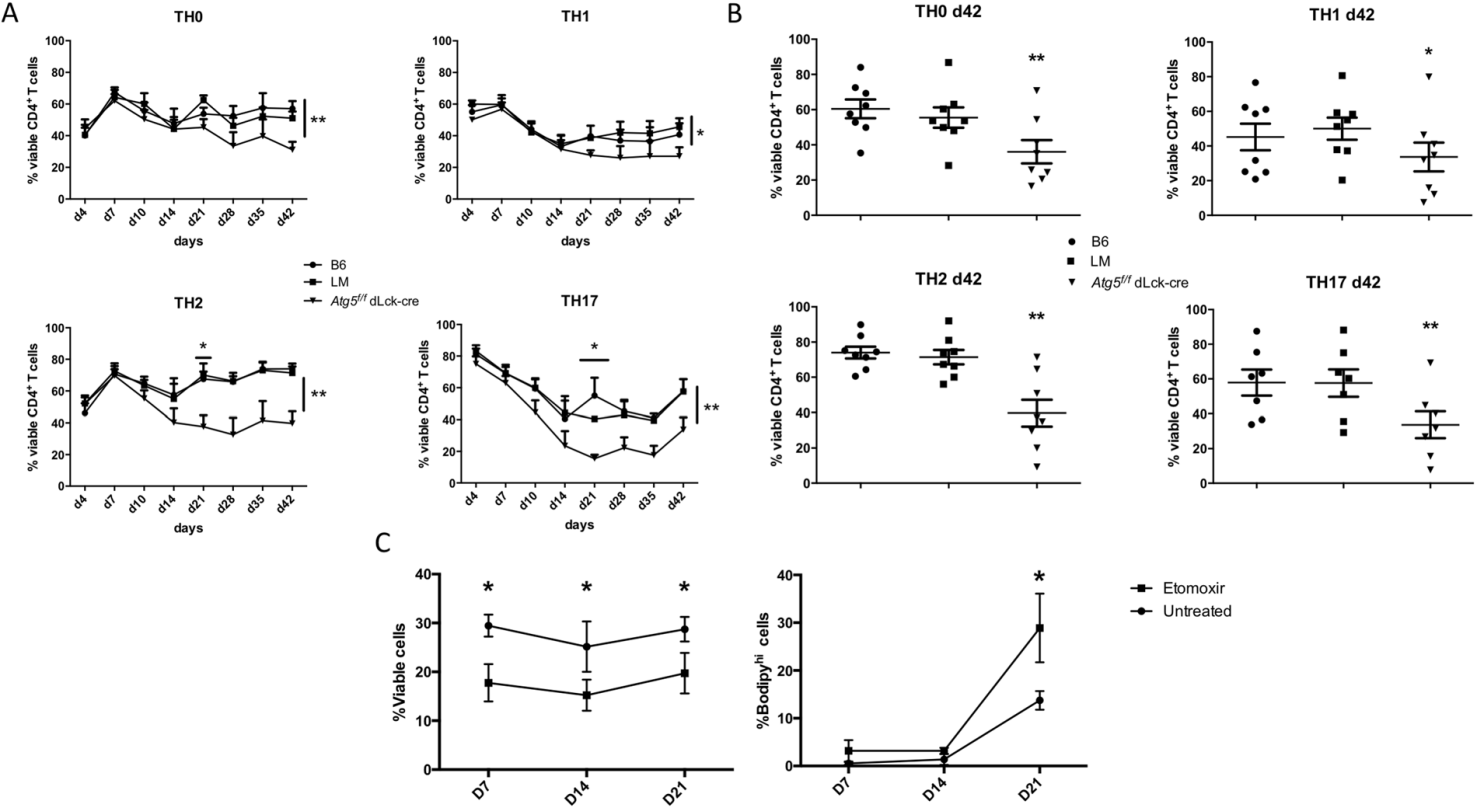
CD4^+^ T cells from C57BL/6 (B6) littermate (LM) and *Atg5*^*f/f*^ dLck-cre mice, were isolated and stimulated by anti-CD3 and anti-CD28 antibodies for 7 days in the presence of polarizing cytokines to differentiate cells into Th0, Th1, Th2 or Th17 cells. Cells were then maintained in IL-7 supplemented medium for additional 35 days. (**A**) Longitudinal study of cell viability by flow cytometry measurement of AnnexinV/propidium iodide (PI) negative cells at indicated days for Th0, Th1, Th2, and Th17 cells. (**B**) Results obtained on individual experiments for cell survival at the end of the protocol, at day 42. Each point represents an individual measure; histograms stand for means and bars represent standard deviation. (**C**) B6 CD4 T cells were stimulated according to the same protocol for 21 days, and treated or not by 200 µM Etomoxir. Cell viability was assessed by measuring Annexin V-/PI- cells. Neutral lipid content was also measured and evaluated by the number of cells with high bodipy mean fluorescence (Bodipy^hi^ cells). *Atg5*^*f/f*^ dLck-cre mice are compared with LM by Mann Whitney U Test to LM mice (n = 8 per genotype). ***p < 0.001, **p < 0.01, *p < 0.05.

**Figure 8:**
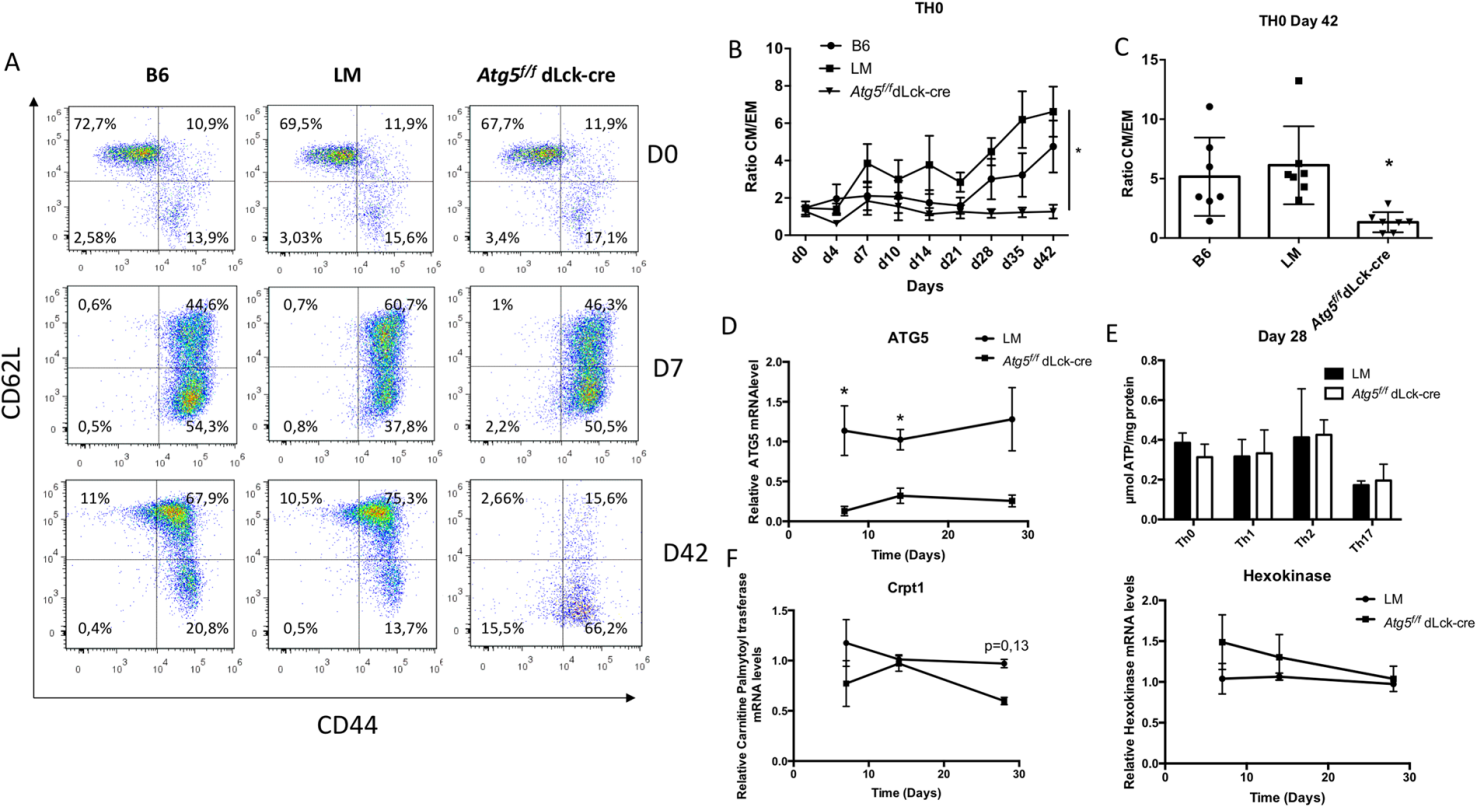
CD4^+^ T cells were isolated from C57BL/6 (B6) littermate (LM) and *Atg5*^*f/f*^ dLck-cre mice and stimulated by anti-CD3 and anti-CD28 Abs for 7 days in the presence of polarizing cytokines to differentiate cells into Th0, Th1, Th2 or Th17 cells. Cells were then maintained in IL-7 supplemented medium for additional 35 days. (**A**) At indicated days (0, 7 and 42) cells were stained to detect CD44 and CD62L expression and analyzed by flow cytometry. A representative experiment is shown for each genotype (B6, LM and *Atg5*^*f/f*^ dLck-cre). Central memory T cells (CM) were defined as CD44^hi^CD62L^hi^, effector memory T cells (EM) were defined as CD44^hi^CD62L^lo^ and naive cells were defined as CD44^lo^CD62L^hi^. *Atg5*^*f/f*^ dLck-cre mice are compared with LM by Mann Whitney U Test to LM mice (n = 8 per genotype). *p < 0.05 (**B**) Longitudinal study of the ratio between percentages of CM T cells and EM T cells among viable cells, measured by flow cytometry, at indicated days for Th0 cells. (**C**) Results obtained on individual experiments for ratio between percentages of CM T cells and EM T cells among viable cells at the end of the protocol, at day 42. Each point represents an individual measurement; histograms stand for means and bars represent standard deviation. (**D**) Quantification of *Atg5* transcripts in the cultured Th0 cells at days 7, 14 and 28 by real-time PCR. One littermate sample is arbitrarily set to 1. n = 4 each genotype except for D28 with n = 2 littermates and n = 4 *Atg5* deficient T cell samples. (**E**) ATP concentration measured on cell lysates for cell cultures at 28 days, with the conditions indicated in D. (**F**) *Hexokinase* and *Carnitine palmitoyltransferase* transcripts, were quantified by real-time PCR, after cDNA generation, on the cultured Th0 cells at days 7, 14 and 28. One littermate sample is arbitrarily set to 1. n = 4 each genotype except for D28 with n = 2 littermates and n = 4 *Atg5* deficient T cell samples.

**Figure 9:**
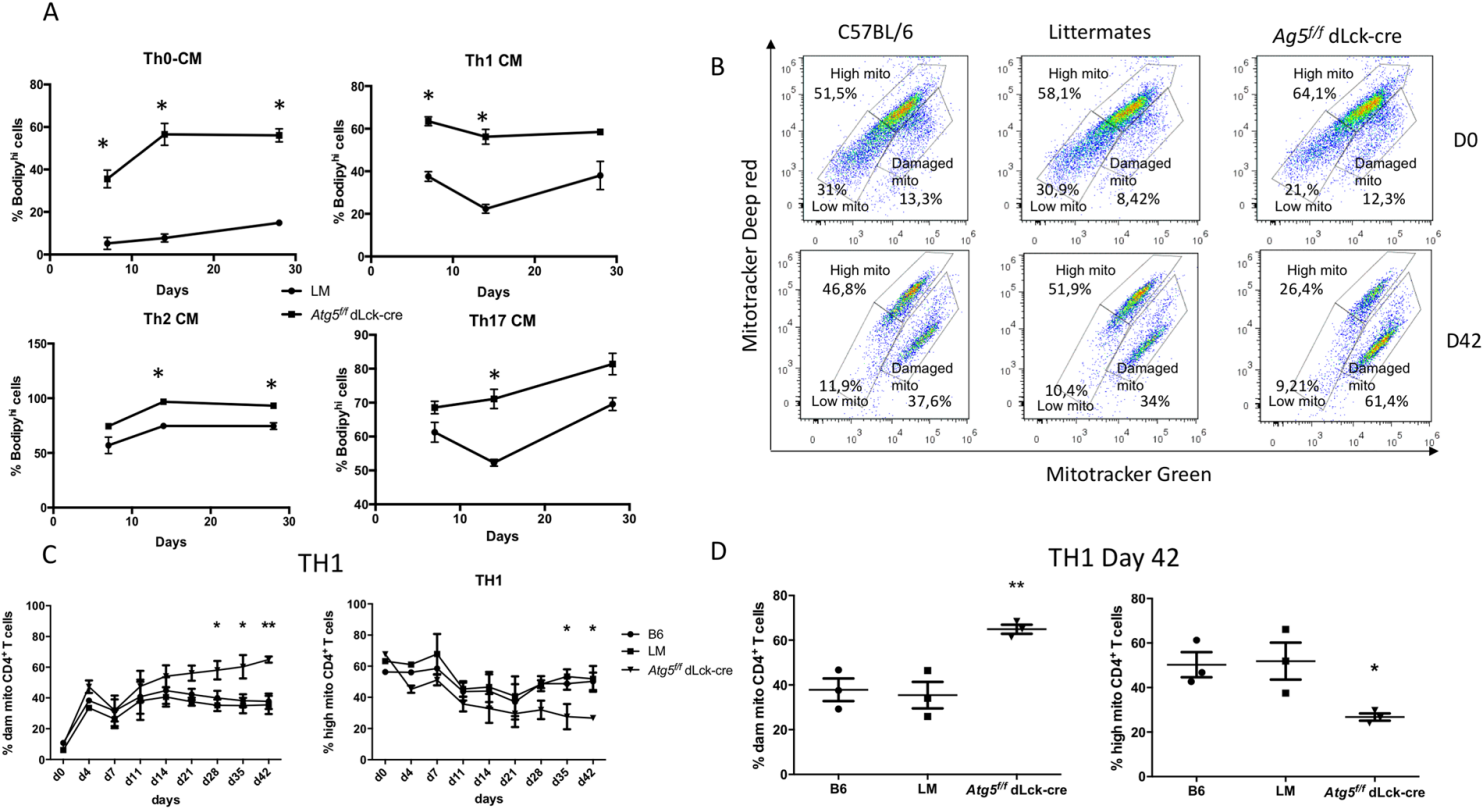
CD4^+^ T cells were isolated from C57BL/6 (B6) littermate (LM) and *Atg5*^*f/f*^ dLck-cre mice and stimulated by anti-CD3 and anti-CD28 Abs for 7 days in the presence of polarizing cytokines to differentiate cells into Th0, Th1, Th2 or Th17 cells. Cells were then maintained in IL-7 supplemented medium for additional 35 days. (**A**) Longitudinal study of bodipy staining intensity. Cells were stained for CD62L and CD44. Cells exhibiting a CM phenotype (CD44^hi^CD62^low^), with a high level of bodipy staining, were quantified at days 7, 14 and 28. (**B**) At indicated days (0 and 42), cells were stained by mitotracker deep red and mitotracker green. Gates could be defined for cells with high load of mitochondria (mitotracker deep red^hi^, mitotracker green^hi^), cells with low mitochondrial content (mitotracker deep red^low^, mitotracker green^low^) and cells with damaged mitochondria (mitotracker green^hi^, mitotracker deep red^low^). A representative experiment is shown. (**C**) Longitudinal study of percentages of CD4T cells with high mitochondria content (right) or damaged mitochondria (left). (**D**) Results obtained on several individual experiments for percentages of CD4^+^ T cells with high mitochondria content (right) or damaged mitochondria (left) at day 42. Each point represents an individual measurement; histograms stand for means and bars represent standard deviation. *Atg5*^*f/f*^ dLck-cre mice are represented and compared with LM by two-way ANOVA (n = 3 for each genotype). *p < 0.05 **p<0,01.

We then aimed at delineating the underlying defects related to autophagy deficiency in CD4^+^ T cell memory. We sorted naive (CD44^lo^CD62L^hi^), effector memory (EM, CD44^hi^CD62L^lo^) and central memory (CM, CD44^hi^CD62L^hi^) T cells from littermate controls or *Atg5*^*f/f*^ dLck-cre mice, by flow cytometry. As shown before (Fig. 1E), no major difference in CM, EM and naive CD4^+^T cell repartition was observed before sorting, in *Atg5*^*f/f*^ dLck-cre mice, in comparison to control mice (Fig. 6A). However, when we performed *Atg5* transcript quantification, we observed that in contrast to naive T cells, EM and CM CD4^+^ T cells from *Atg5*^*f/f*^ dLck-cre mice expressed *Atg5* transcript levels closer to control mice (Fig. 6B). This could reflect a selection *in vivo* of cells expressing normal levels of ATG5, *i.e*. incompletely deleted by the cre activity. This observation supports the notion that autophagy is integral to the maintenance of memory. Interestingly we observed in naive T cells, a higher level of neutral lipids, in the absence of autophagy (Fig. 6C,D). This feature is less pronounced in memory cells, that partially escaped *Atg5* deletion according to our previous observation. This suggests that the regulation of lipid stores might be important for memory T cell survival. Interestingly, the overall levels of lipids in memory cells is higher than in naive cells. It is known that memory cells display a different metabolism than naive cells, favouring FAO. We thus quantified the levels of hexokinase A and palmitoyl-carnitine transferase enzyme transcripts (Fig. 6E). We were not able to detect any difference in the levels of these enzymes, either in naive or in memory cells. This could however be due to escape from the deletion in memory cells between littermate and *Atg5*^*f/f*^ dLck-cre mice or indicate that the disequilibrium in neutral lipid stores in the absence of autophagy has no major consequence on the balance between glycolysis and FAO. In accordance with this latter hypothesis, we showed that 7-day activation of T cells deficient for autophagy does not lead to more lactate production than control T cells (Figure S6). To overcome the selection of autophagy-competent cells *in vivo*, we generated memory cells *in vitro*, to assess the role of autophagy in CD4^+^ T cells. We magnetically-sorted CD4^+^ T cells from LM controls or *Atg5*^*f/f*^ dLck-cre mice. We first stimulated them with anti-CD3 and anti-CD28 antibodies (Abs). In accordance with our previous findings, if no other survival signal was added, we did not detect any difference in terms of survival either in the absence or in the presence of autophagy in CD4^+^ T cells, in the days that followed this initial activation (Figure S7). As previously mentioned, cells initially purified showed no escape from *Atg5* deletion by the cre recombinase (Figure S3). We then aimed at polarizing cells with cytokines, and culturing them during a long period of time, in the presence of IL-7. The latter cytokine is known to drive and to maintain a memory phenotype. Interestingly, during the first days following activation, the survival rate was irrespective of CD4^+^ T cell genotype (Fig. 7A). However, from 21 days on after the start of the culture until the end of the experiment at day 42, in all the tested polarization conditions, a significant decreased survival of CD4^+^ T cells was seen with *Atg5*^*f/f*^ dLck-cre mice (Fig. 7A,B). Interestingly, the carnitine-palmitoyl transferase inhibitor etomoxir, also led in our system to an overall decrease of cell viability of wild-type CD4^+^ T cells, in IL-7 supplemented cultures (Fig. 7C). Moreover, this long-term etomoxir treatment leads to an accumulation of neutral lipids, reminiscent of what is observed in *Atg5*^*f/f*^ dlck-cre CD4 + T cells. This suggests that autophagy could play a role in the mobilization of lipids in long-term T cell cultures or in limiting the toxicity of lipid storage. During the *in vitro* differentiation of memory cell, we also monitored the acquisition of the memory phenotype by CD44 and CD62L staining. We showed again that initial activation of CD4^+^ T cells is poorly impacted by the absence of autophagy. Indeed, the proportion between central memory (CM) and effector memory (EM) cells was comparable between cells purified from wild type, LM control, and *Atg5*^*f/f*^ dLck-cre-mice (Fig. 8A,B). However, after day 21, *Atg5*-deficient CD4 T cells failed to efficiently maintain a CM phenotype, compared to controls, as shown by the stagnation of the CM/EM ratio (Fig. 8A–C). The results shown here for Th0 cells, were observed in the other polarization conditions except for Th1 cells, where statistical significance was not reached (Figure S8). These results support the hypothesis that autophagy allows the survival of CM T cells. Contrary to the *in vivo* findings, CM T cells *in vitro* do not escape *Atg5* deletion, as shown by the absence of *Atg5* transcript enrichment after quantification (Fig. 8D). We could then assess bioenergetic levels and strategy for energy production, in memory cells deficient for autophagy, in a more reliable way than in *ex vivo* cells. No difference in overall ATP cellular level was identified in ATG5-deficient memory T cells at day 28 (Fig. 8E, same results at day 21, results not shown). Same as in *ex vivo* situation, we found again no obvious difference in glycolysis enzyme transcript level in Th0 cells (Fig. 8F). A decrease, not reaching significance with the relatively low number of samples, is observed for *cprt1*. In line with this observation, we found a strong difference in lipid load during the differentiation of memory cells *in vitro*, more striking than the results *in vivo*, where some cells still express *Atg5* (Fig. 9A). All these results underline that, although autophagy controls lipid stores in memory T cells, cell death observed in ATG5-deficient cells is not primarily due to a major energy breakdown but could be linked to a defective FAO. We moreover observed that from day 0 to day 42, CD4^+^ T cells maintain a high load of mitochondria (Fig. 9B–D). We also observed a significantly increased population of T cells with damaged mitochondria, meaning cells with depolarized mitochondria, together with a failure to maintain high loads of healthy mitochondria. Collectively, these results suggest that autophagy is an important factor in the survival of memory CD4^+^ T cells, mainly through the regulation of mitochondrial homeostasis. Autophagy activity could be important to limit the production of toxic compounds released by mitochondria, or accumulated as neutral lipids.

## Discussion

In this work, we generated a model with autophagy deletion in T cells, only from the mature stage on. We were thus able to bring new information about the precise role for autophagy in T cell homeostasis. As a matter of fact, models described previously^5–9,15^ could not exclude a negative impact of autophagy deletion during T cell development, leading to the acute impaired survival and function that have been reported. The work by Jia *et al*., also investigated the impact of autophagy deficiency triggered only at the mature stage^15^ but with a different model. They used an inducible *Atg3* deletion model, with estrogen receptor promoter-mediated deletion. They described defects during long-term cultures after tamoxifen treatment *in vitro*, thus in mature T cells. However, this model did not allow to study the *in vivo* behaviour of autophagy-deleted T cells. Our aim was to fill this gap of knowledge and analyse *in vivo* the consequences of autophagy inhibition in T cells in the periphery, excluding misinterpretations due to developmental issues. We report here that CD4^+^ T cells are poorly sensitive to autophagy impairment in the mature stage, contrary to CD8^+^ T cells. It has been reported previously that mitophagy was a major actor for T cell survival. Indeed, thymocytes exhibit a high mitochondrial load^4,7^. During thymus egress of differentiated cells, mature T cells lose a considerable part of their mitochondrial load. Interestingly, we report here that this decrease in mitochondrial load is more pronounced in CD8^+^ T cells than in CD4^+^ T cells. Moreover CD8^+^ T cells produced more mitochondrial ROS in the absence of autophagy, which could explain their survival defect in this context. In relation to these findings, we found *in vitro* that CD4^+^ T cell activation, survival and proliferative capacity were not affected by the impairment of autophagy. These results may seem surprising considering the results published by Jia and colleagues showing that degradation of p27, favouring proliferation under TCR stimulation, is mediated by autophagy^16^. In their work, however, most *in vivo* experiments focused on CD8^+^ T cells, in which autophagy seems to be more required for homeostasis. Moreover, experiments monitoring CD4^+^ T cells were performed with deletion early during development (by the use of the proximal Lck promoter). Collectively these results led to the conclusion that autophagy is not required for naive CD4^+^ T cell homeostatic survival and short-term activation, in contrast to CD8^+^ T cells.

*In vivo* experiments with *Atg5*^*f/f*^ dLck-cre mouse model showed that autophagy was not required for short-term antibody response against a T-dependent antigen. Moreover, total IgG and IgM levels were found to be normal in *Atg5*^*f/f*^ dLck-cre mice, confirming that no major humoral immunosuppression occurs. Interestingly, however, we observed that long-term immune response was compromised in the absence of autophagy in CD4^+^ T cells. As we ruled out increased senescence of the CD4^+^ T cell compartment with *Atg5* deficiency, we hypothesized that memory CD4^+^ T cells were selectively impacted by the absence of autophagy. Indeed, we observed that antigen-experienced CD4^+^ T cells transferred to a naive host were unable to recapitulate the memory effect. We also evidenced a selection of autophagy-competent T cells in the central memory T cell compartment, showing the preferential importance for autophagy in this cell population in comparison to naive T cells. Moreover, memory CD4^+^ T cells differentiated *in vitro* exhibited decreased survival. These findings are reminiscent of previous studies indicating that autophagy is integral to memory CD8^+^ T cell survival. Using transfer experiments with autophagy deficient CD8^+^ T cells^11^, or mouse strains with autophagy deletion occurring only at the cytotoxic stage^12^, it has been previously shown that autophagy allowed the maintenance of the memory CD8 T cell compartment, and was mandatory for influenza memory cytotoxic response. Puleston and colleagues further showed that memory CD8^+^ T cells required autophagy for limitation of mitochondrial load and generation of ROS leading to increased apoptosis. We also found a deregulated mitochondrial pool in autophagy-deficient memory CD4^+^ T cells showing that they require mitophagy for proper removal of damaged mitochondria. We further discovered that this process seems more important for CM T cell survival than for EM T cells. Memory T cells have been shown to particularly rely on energy produced by mitochondria, such as FAO, for their long term survival^2^. We cannot rule out either an importance of proper ER content control by autophagy as suggested by Jia *et al*.^15^.

We also identified for the first time a deregulated lipid storage in the absence of ATG5. Interestingly, lipid stores are more important in memory T cells, which can be put in line with the higher FAO activity of these cells compared to naive cells. Such a deregulation was also observed *ex vivo*, but only in naive cells, that did not escape ATG5 deletion. The *in vitro* experiments show that although memory cells exhibit aberrant mitochondrial populations and lipid stores, their metabolism seems unchanged. Indeed, no major energy breakdown was noticed in the absence of ATG5, nor unbalanced glycolysis/FAO usage in comparison to control cells. We however observed that treatment by the FAO inhibitor etomoxir, in long-term IL-7 supplemented cultures, leads to an increased CD4^+^ T cell death and an accumulation of lipid stores, resembling the phenotype of *Atg5*^*f/f*^ dLck-cre mice. This suggests that autophagy could facilitate memory CD4^+^ T cell survival by regulating lipid turnover, but not for the generation of energy. We thus propose that the control of mitochondria and lipid stores by autophagy is particularly important to remove toxic compounds as ROS or excess lipids. Interestingly, high lipid loads are linked to cell death, especially via ER stress. This phenomenon may be more pronounced when there is an aberrant lipid oxidation due to mitochondria-derived ROS, that might take place in ATG5 deficient-memory CD4 T cells. Indeed, lipophagy has been shown to protect several cell types from cell death^17^. This aspect deserved further studies.

In conclusion, we report here the crucial importance of autophagy in CD4^+^ T cell-related immune responses. Added to the previously described role of autophagy in CD8^+^ T cell memory response and thus in cytotoxic effector responses, our results lead us to conclude that optimal antibody responses require autophagy in T cells. These results can also be put in line with the described roles for autophagy in memory B cells and plasma cell long-term maintenance^18–22^. Thus, in support of recent data^11,12^, our findings reinforce the idea that increasing autophagy could optimize the efficiency of humoral responses at several levels. Moreover, inhibiting autophagy could limit the chronicity of systemic autoimmune responses, and this not only at the level of the B cell lineage as recently described^21^, but also by limiting the persistence of pathogenic autoreactive CD4^+^ T cells.

## Material and Methods

### Mice

*Atg5*^*f/f*^ mice were kindly provided by Prof. N. Mizushima^23^. They were crossed with *distal Lck (dLck)-*cre mice for mature T-cell specific deletion^14^. *Atg5*^*f/f*^ dLck-cre mice were compared with either C57BL/6 mice, or littermates (B6 *Atg5*^*f/*+^ dLck-cre mice or *Atg5*^*f/f*^ mice). Genotype of mice was determined for *Atg5* alleles by PCR as described in^21^. Presence of the cre transgene was assessed with the following primers pairs: cre-1 5′-ATGGTGCCCAAGAAGAAGAG-3′; cre-2 5′-CAGGTGCTGTTGGATGGTCT-3′. Animal experimentation was performed according to guidelines of the local Institutional Animal Care and Use Committee (CREMEAS). Authorization for the presented experimentations was given by CREMEAS (reference AL/07/07/01/13).

### Real-time PCR

Total RNA was purified from 5 × 10^6^ T cells with RNeasy Mini Kit or from 10^5^ cells with the RNeasy Micro Kit (Qiagen, Courtabeuf, France, 74103 or 74004) according to the manufacturer’s instructions. Samples were treated by DNAse (Qiagen, 79254) and RT was performed with the Maxima first strand synthesis (ThermoFisher, Illkirch, France). cDNA (10 ng) were used for real-time PCR (RT-PCR) on StepOne apparatus (Thermo-Fisher). *Atg5, Hexokinase (Hk2), Carnitine Parlmitoyl Transférase (cpt1a)* and *Gapdh* expression was assessed using Taqman Gene Expression Assays (ThermoFisher: Mm00504340_m1, Mm99999915_g1, Mm00443385_m1, MMm01231183_m). *Atg5* mRNA levels were quantified by defining ΔCT (CT *Gapdh*− CT “tested gene” where CT is ‘Cycle Threshold’) and ΔΔCT (ΔCT sample−ΔCT of one C57BL/6 mouse sample used for each plate). Values indicated correspond to 2^−ΔΔCT^. The value obtained for 1 control sample in each plate is set to 1.

### Flow cytometry and cell sorting

The following antibodies were used for flow cytometry analysis. For surface stainings: allophycocyanin (APC)-cyanine 7-labelled anti-mouse TCR-β (clone H57-597, BD Biosciences 553139), phycoerythrin (PE)-labelled anti-mouse CD62L (clone MEL-14, BD Biosciences 553151), fluorescein isothiocyanate (FITC), APC or peridinin chlorophyll (PerCP) cyanine 5.5-labelled anti-mouse B220 (clone RA3-6B2, BD Biosciences, 553087, 553092, and 552771), FITC-labelled anti-mouse CD3ε (clone 145-2C11, BD Biosciences 553061), APC cyanine7, PE or PerCP cyanine 5.5, APC-labelled anti-mouse CD4 (clone GK1.5,552051, eBioscience 12-0041 or clone RM4-5, BD Biosciences, 550954, 553051), PerCP cyanine 5.5-labelled anti-mouse CD8 (clone 53-6.7, BD Biosciences, 551162), APC cyanine 7 or FITC-labelled anti-mouse CD44 (clone IM7, BD Biosciences 559250, 103027, 553133), PE-labelled CD127 (IL-7R, clone A7R43 eBioscience 12-1271-82). For intracellular stainings: PE-labelled anti-mouse IL-4 (clone BVD4-1D11, BD Biosciences, 554389), PE-labelled anti-mouse IL-17A (clone eBio17B7, 12-7177-81), FITC-labelled anti-mouse IFN-γ (clone XMG1, BD Biosciences, 554411), Alexa-647-labelled anti-mouse/human TBET (clone O4-46, BD Biosciences, 561267), PE cyanine7-labelled anti-mouse GATA3 (clone L50-823, BD Biosciences, 562683), PerCP cyanine 5.5-labelled anti-mouse RORγT (cloneQ. 31-378, BD Biosciences, 560405), GL7-FITC (clone GL7, Biosciences 553666), anti-CD95 AF647 (clone JO2, BD Biosciences 563647), anti-PD1 (clone J43, BD Biosciences 551892), anti-CXCR5 APC (Clone 2G8, BD Biosciences 560615) anti-CD138 APC clone 281-2, BD Biosciences 558626). Splenocytes, thymocytes or lymph node cells were stained with indicated Abs and Fc blocking Ab CD16/CD32 Ab (clone 2.4G2, BD Biosciences, 553142) as described in^21^. After surface staining, in some cases, fixation and permeabilization set (eBioscience; 88-8824-00) was used to detect intracellular molecules. Cells were incubated 30 min in the fixation buffer, followed by a 10 min-incubation with anti-CD16/32 antibody in the buffer furnished by supplier and were finally stained for 45 min with Abs indicated above. Between each step, cells were washed with supplier’s buffer. Mitochondrial load and membrane potential were assessed by staining cells with respectively mitotracker green and deep red dyes (Fisher Scientific, Pittsburgh, PA, USA; M-7514 and M22426) as described in^21^. Mitochondrial ROS production was assessed by Mitosox staining according to manufacturer’s instruction (Fischer Scientific, M36008). Lipid storage was monitored with Bodipy staining (Fisher Scientific, Illkirch, France) in PBS for 20 minutes at 37 °C. Cell survival was assessed using FITC or APC-labelled Annexin V (BD Bioscience, 556419, 550475) in combination with propidium iodide (PI) at 1 µg/mL (Sigma-Aldrich, Saint-Quentin, France; P4170) or 7-aminoactinomycin D (7-AAD, BD Pharmingen, 559925) following the indications from the suppliers. Samples were processed on a Gallios flow cytometer (Beckman Coulter, Fullerton, CA, USA) and data were analysed with FlowJo software v10 (FlowJo LLC, Ashland, OR, USA). Proliferation indexes were determined according to the “proliferation modelling” tool provided by FlowJo Software. Naive, effector memory, central memory CD4+ T cells were sorted from mouse spleens at the cytometry facilities of IGBMC (Institute for genetics, molecular and cellular biology, Illkirch, France) after TCRβ, CD4, CD62L and CD44 staining. Purity of each fraction was >98%.

### ATP and lactate quantification

Briefly, 1.10^5^ cells were lysed in the following buffer: 25 mM Tris-HCl, 150 mM NaCl, pH 7.5, 0.1% (v/v) Triton X-100 in the presence of protease inhibitors. ATP cellular levels were quantified with the ATP determination kit (Fisher Scientific, France). The results were normalized relative to the total protein content measured by BCA kit (Pierce). Lactate was quantified on supernatant from 7-day cultures of purified CD4+ T cells in the aforementioned conditions. The Lactate-Glo kit from Promega (Charbonnières-les-bains, France) was used according to manufacturer’s instructions.

### Western Blot

The following antibodies were used for immunoblots: ACTB (Santa Cruz Biotechnology, clone C4, sc-47778), MAP1LC3 (LC3, MBL, clone 51–11, ref M115–3) and ATG5 (Rabbit Polyclonal, Novus, NB110-53818). In indicated conditions, cells were previously treated by lysosomal protease inhibitors E64d and pepstatin A (5 µg/mL each, Sigma-Aldrich, P5318 and E8640 respectively). When indicated, cells were stimulated with hamster anti-mouse CD3ε (5 µg/mL, clone 145-2C11, 55305, BD Pharmingen), hamster anti-mouse CD28 (5 µg/mL, and clone 37.51, 553294, BD Pharmingen), phorbol-12-myristate 13-acetate (PMA, 50 ng/mL, Sigma) or ionomycin (1 µM, Sigma). Immunoblots were processed as described in^21^.

### Immunizations

Intra-peritoneal (i.p.) injections were performed on day 1 and 10, with mice aged 8-20 weeks. A third immunization was performed 12 weeks after the first one. Mice were bled on days 5 and 15, on weeks 8 and 12 + 5 days. Mice were immunized by OVA (100 µg, Sigma), at first injection in CFA (complete Freund’s adjuvant, Sigma) and at second and third injections in IFA (incomplete Freund’s adjuvant, Sigma). Detection of anti-OVA IgM and IgG Abs was assessed by ELISA.

### Antibody detection by ELISA

General procedure for ELISA was previously described^21^. Serum levels of total IgG and IgM were determined in immunized mice using the IgM/IgG quantification kit from Bethyl laboratories following manufacturer’s indications (Bethyl, Montgomery, TX, USA, E90-101/E90-131). Concentrations were determined with standard curves. Anti-OVA specific Abs were titrated by serial dilutions of serum. Titers correspond to the inverse of last dilution with optical density ≤ 0.2. IgG and IgM absolute quantifications were performed after comparison to a standard.

### Cell isolation and culture

Spleens were collected from B6, dLck-cre *Atg5*^*f/f*^ mice or from littermates. Splenic CD4^+^ or CD8^+^ T were isolated with the Dynabeads untouched mouse CD4 or CD8 cells isolation kit (ThermoFisher Scientific, Illkirch, France, respectively 11415D and 11417D). Cells were >90% pure according to TCRβ^+^/CD4^+^/B220^−^ or TCRβ^+^/CD8α^+^/B220^−^ population quantifications by cytometry. When indicated, cells were stimulated with hamster anti-mouse CD3ε (5 µg/ml, clone 145-2C11, 553057, BD Pharmingen), hamster anti-mouse CD28 (5 µg/ml, and clone 37.51, 553294, BD Pharmingen), PMA (50 ng/mL, Sigma) or ionomycin (1 µM, Sigma). Cell proliferation was assessed by staining cells with carboxyfluorescein succinimidyl ester diacetate (0.5 µM, CFSE, Sigma) before stimulation. When indicated, proliferation indexes were calculated with the tool provided by FlowJo and corresponds to the total number of divisions divided by the number of cells that went into division.

### CD4 T cell polarization and long-term culture

After isolation, splenic CD4 T cells were cultured at 37 °C, 5% CO_2_ in complete medium (RPMI 1640 medium, 10% FCS, 10 mg/mL gentamycin, 10 mM HEPES and 0.05 mM β-mercaptoethanol, all purchased from Lonza BioWhittaker) supplemented with the according cytokine/antibody cocktail for each T helper cell subset. For the non-polarized condition (Th0), cells were only stimulated with anti-mouse CD3ε and CD28 Abs (clone 145.2C11, 553057, and clone 37.51, 553294, see cell isolation and cell culture for the concentrations). In addition to the anti-CD3/CD28 Ab stimulation, anti-mouse IL-4 Ab (10 µg/mL, clone 11B11, 554432) and recombinant mouse IL-12 (2 ng/mL, p70, 554592) were added, for Th1 polarization, anti-mouse IFN-ɣ Ab (10 µg/mL, clone XMG1.2, 554408) and recombinant mouse IL-4 Ab (15 ng/mL, 550067) for Th2 polarization and finally anti-mouse IL-4 Ab, anti-mouse IFN-ɣ Ab, recombinant mouse IL-6 (10 ng/mL, 554582) and recombinant mouse TGF-β1 (1 ng/mL, R&D Systems, 7666-MB-005), for Th17 polarization. The cells were cultured at a density of 10^5^ cells/well in a 96-well plate for four days. Then, cell polarization was assessed by flow cytometry, cell medium was changed and recombinant IL-7 (5 ng/mL, 407-ML-005, R&D Systems) was added every 3–4 days and the medium changed every 7 days. At these time points, cells were counted, and cell number was adjusted to 10^5^ cells/well. The CD4 T cells were maintained in culture for 42 days. Cytokines and Abs used were purchased from BD Biosciences, except if otherwise specified. In some experiments, cells were treated by 200 µM Etomoxir (Sigma-Aldrich, E1905) for 21 days.

### Adoptive transfer

The donor mice (B6, dLck-cre *Atg5*^*f/f*^ mice or littermates) were immunized with 100 µg OVA/CFA injected i.p. Four weeks later, the mice were sacrificed and CD4^+^ T cells were isolated from the spleen of each mouse. Memory B cells were also isolated from the spleen of one B6 immunized mouse. Positive memory CD4 T cell isolation was accomplished using the CD4 (L3T4) MicroBeads cell isolation kit (130-049-201; Miltenyi Biotec, Bergisch-Gladbach, Germany). Preparations contained more than 85% CD4^+^/CD8α^−^/B220^−^ cells as checked by flow cytometry (~20% were however neither CD8^+^ nor B220^+^). Negative isolation of memory B Cells was performed with the CD43 (Ly-48) MicroBeads cell isolation kit (130-049-801, Miltenyi) following manufacturer’s recommendations. Resulting CD4^−^/CD8α^−^/B220^+^ B cells were >90% pure. CD4^+^ T cells and B cells were co-injected in recipient B6 mice in the proportion of 2 × 10^6^/2 × 10^6^ cells respectively. Seven days after adoptive transfer the recipient mice were immunized with 100 µg OVA/CFA and 7 days later they were bled. Detection of anti-OVA IgM and IgG response was assessed by ELISA.

### Statistical analysis

Data were analyzed with Prism v6 (GraphPad Software Inc, La Jolla, CA USA). Groups were compared by unpaired tests and considered significantly different when p < 0.05. When sample size was sufficient to assume comparable normal distribution (Kolmogorov-Smirnov Test), unpaired t was performed. Otherwise the unpaired non-parametric Mann-Whitney U Test was used. Alternatively, when n <3, we performed two-way ANOVA with Tukey Post-Hoc test.

## Electronic supplementary material

Supplementary Figures
