# Supplementary material for: CD4 T cell autophagy is integral to memory maintenance
Source: Sci Rep. 2018 Apr 13;8:5951. doi: 10.1038/s41598-018-23993-0 (PMC5899169; doi:10.1038/s41598-018-23993-0)
Supplement: Supplementary file 1 — Supplementary Figures [file 41598_2018_23993_MOESM1_ESM.pdf]

CD4 T cell autophagy is integral to memory maintenance

Diane Murera*, Florent Arbogast*, Johan Arnold, Delphine Bouis, Sylviane Muller and Frédéric Gros

Co-first authors

Figure S1: Deletion of ATG5 in peripheral CD4+ T lymphocytes

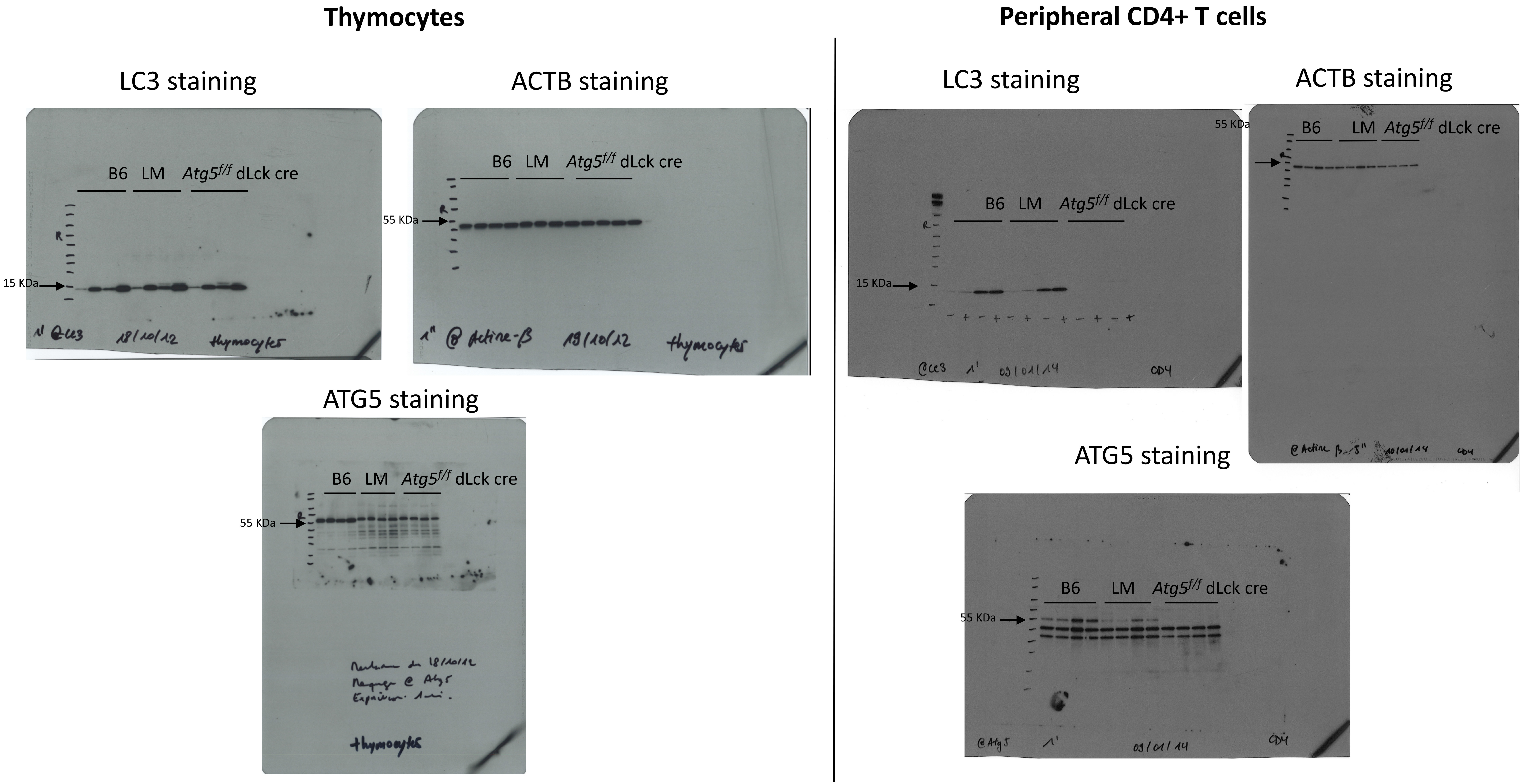

Figure S2: Autophagy is dispensable for CD4 T cell homeostasis in lymph nodes

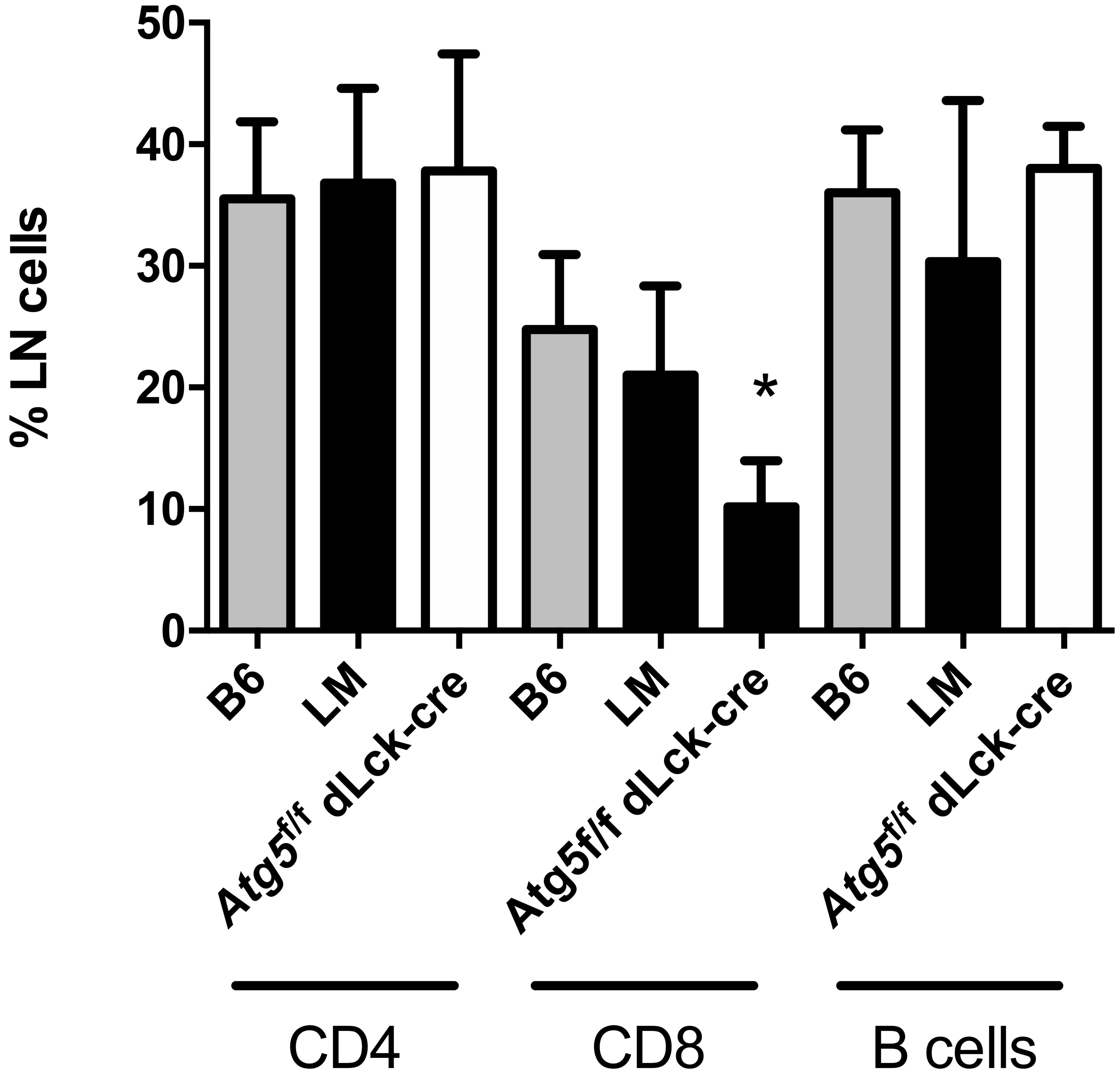

Figure S3: *Atg5* deletion is equally efficient in CD4 and CD8 T cells from *Atg5^{f/f}* dLck cre mice

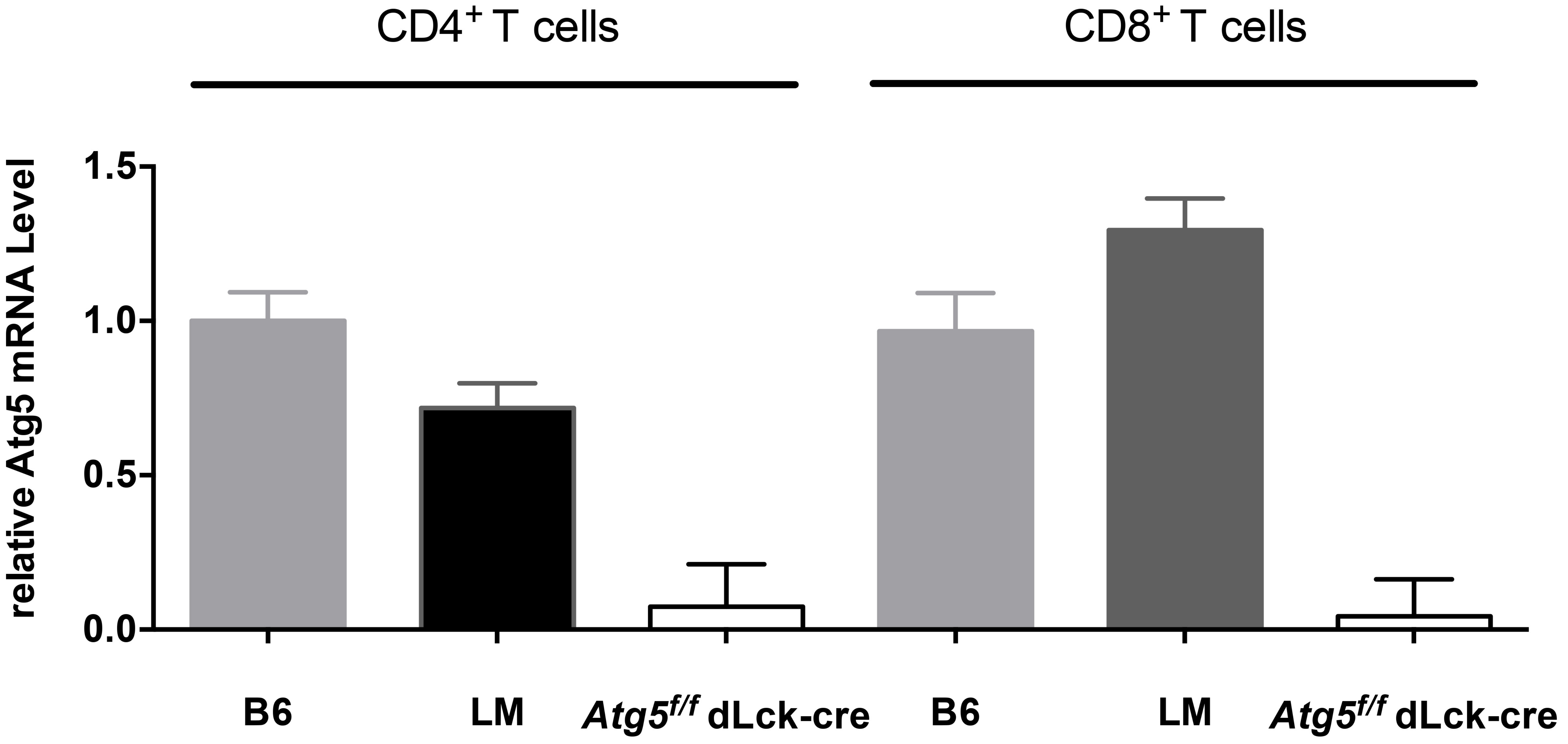

Figure S4: The normal proliferative capacity of CD4 T cells in the absence of autophagy is not due to a selection of ATG5-expressing cells

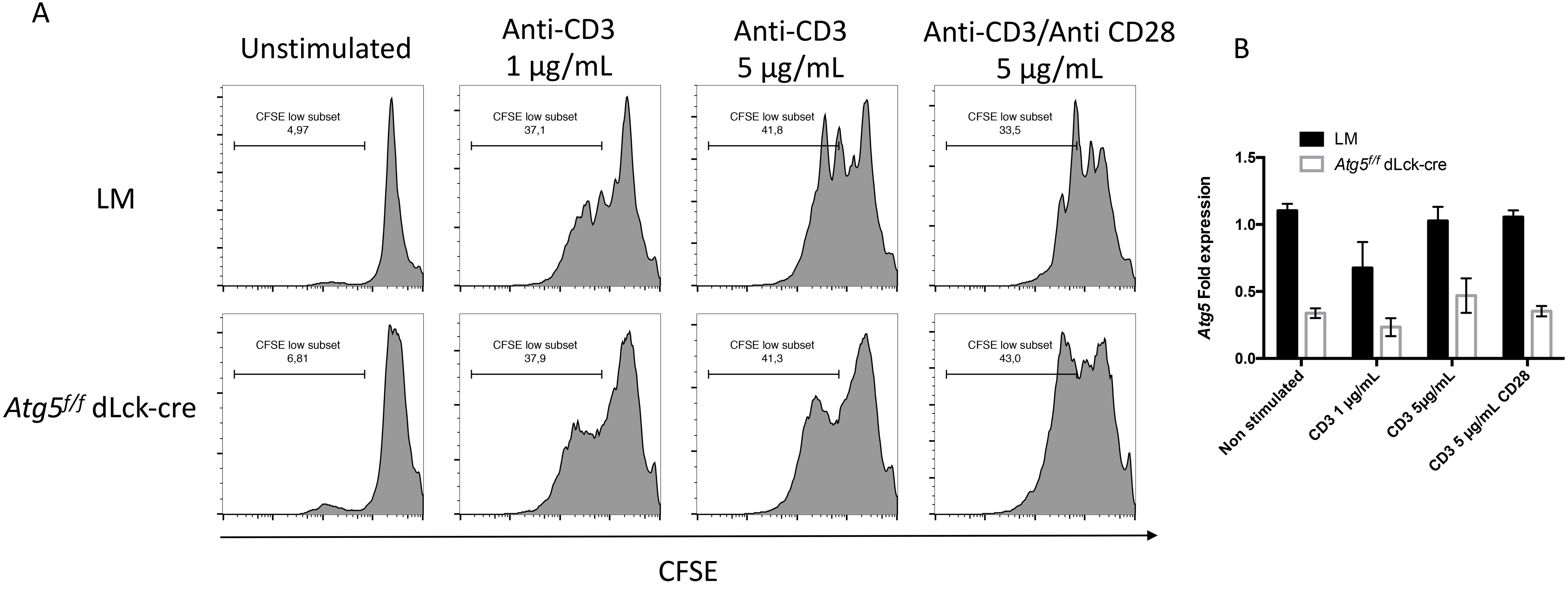

Figure S5: T cell autophagy deficiency affects the long-term humoral response, without global decrease in TFH cells

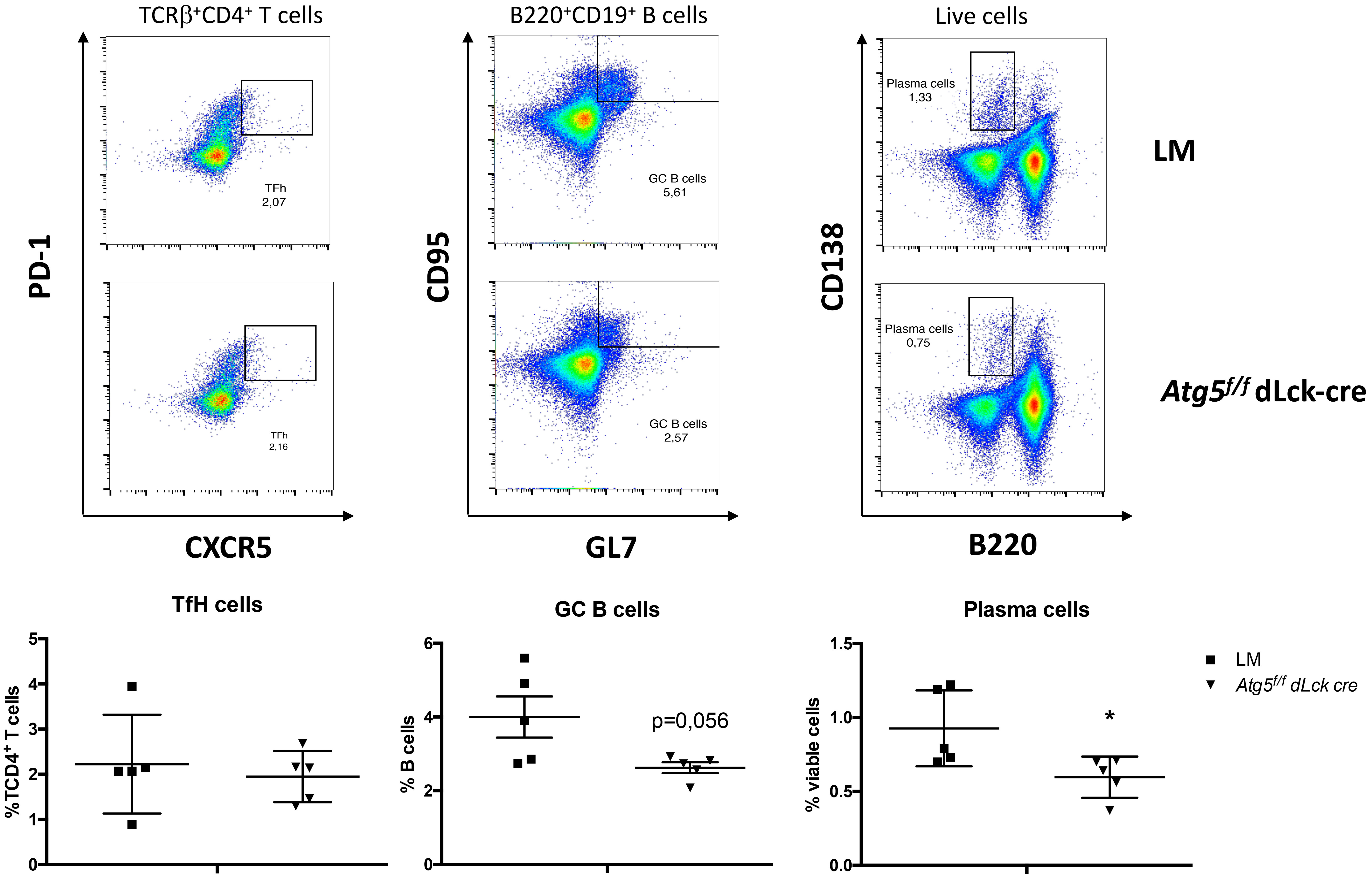

Figure S6: autophagy deficiency in CD4 T cells does not lead to an enhancement of glycolytic activity

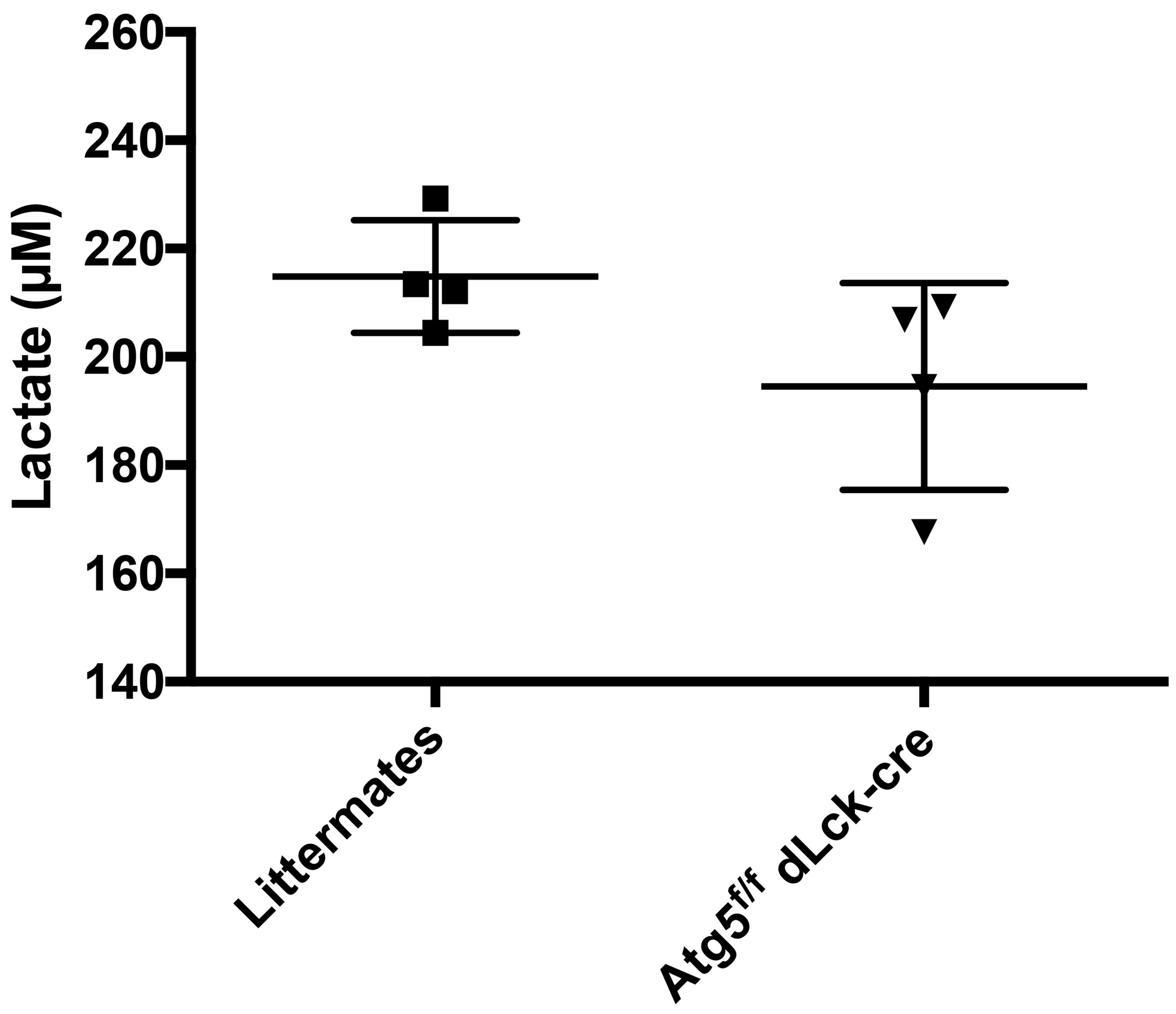

Figure S7: Autophagy is dispensable for survival after TCR stimulation in the absence of IL-7

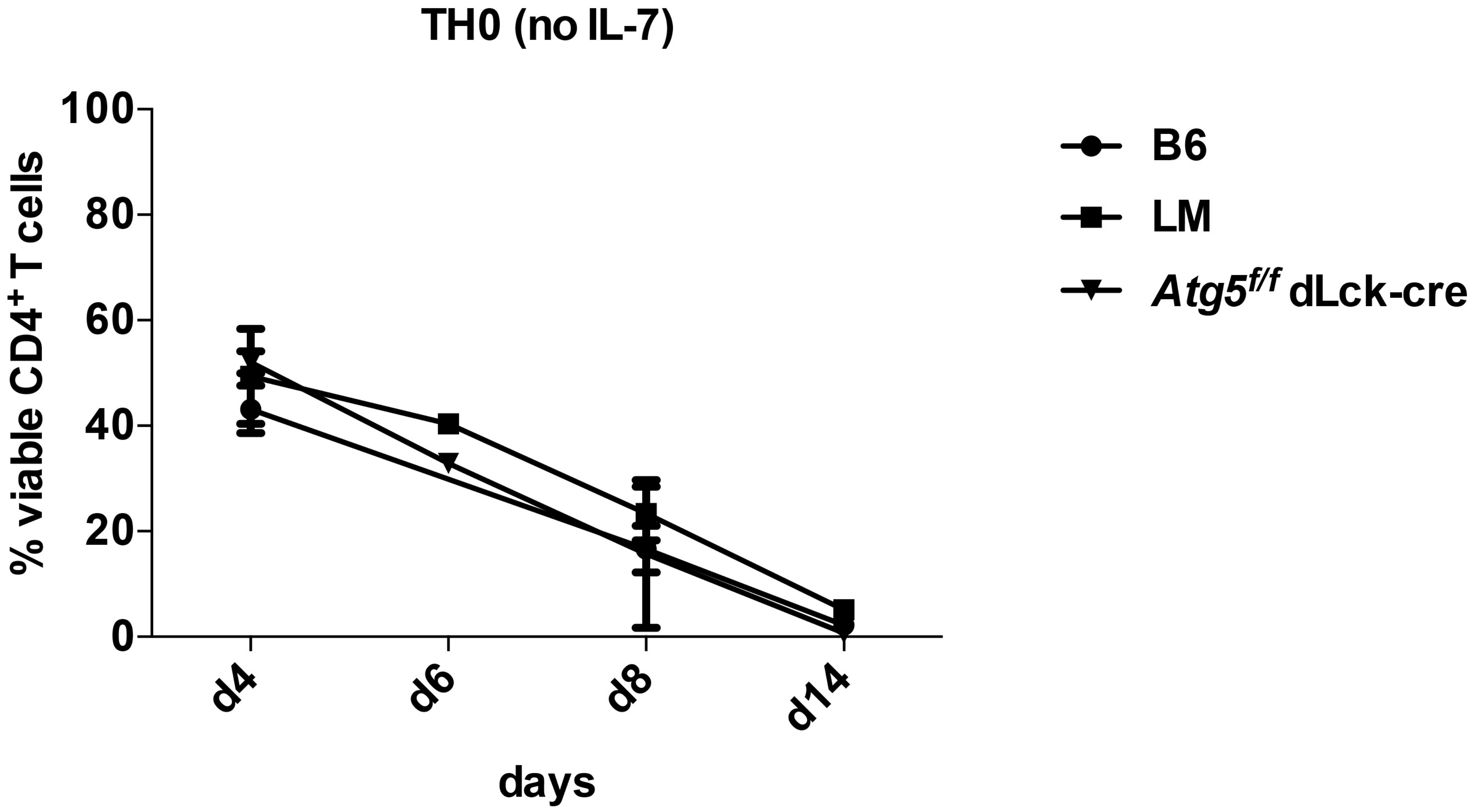

Figure S8: Autophagy is necessary for polarized memory CD4 T cell survival

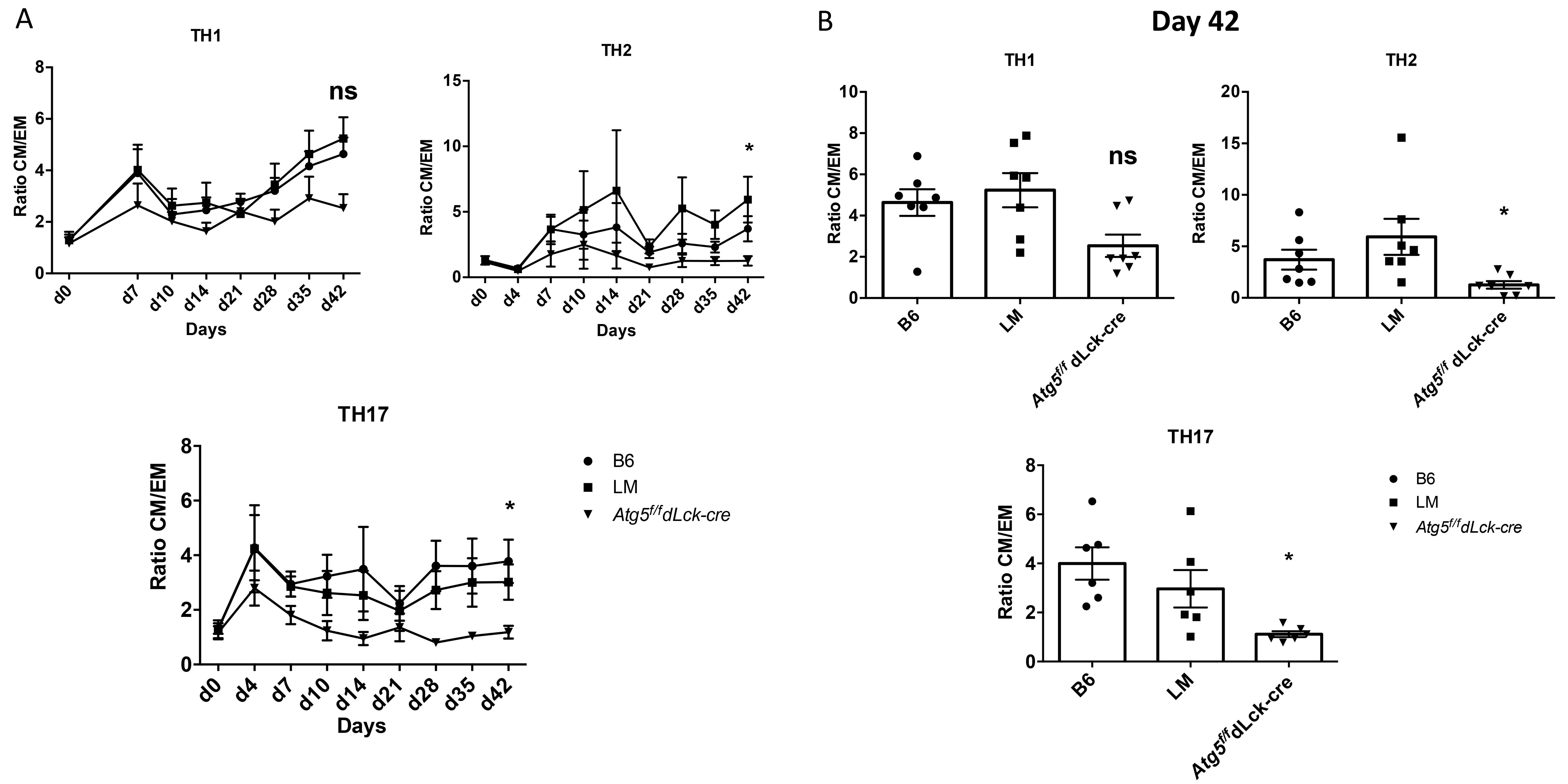

Legends to supplementary Figures

Figure S1

Thymocytes or CD4+ peripheral T cells were isolated from spleens of wild type C57BL/6 (B6) mice, littermate (LM) or *Atg5*$^{f/f}$ dlck-cre mice. Cells were stimulated or not by 50 ng/mL PMA and 1 µg/mL ionomycin for 18 hours. During the last 4 hours of stimulation, cells were treated or not by pepstatin A and E64d. Cell lysates were then processed by SDS-PAGE and blotted against ATG5 and LC3. Representative experiment of at least three replicates. Images of figure 1A are crops from these scans.

Figure S2

Histograms showing the percentages of $CD4^+$ T cells ($TCR\beta^+CD4^+$), $CD8^+$ T cells ($TCR\beta^+CD8\alpha^+$) and B cells ($TCR\beta^-B220^+$) among lymph node cells, assessed by flow cytometry. C57BL/6 (B6), Littermate (LM), *Atg5*$^{f/f}$ dLck cre mice are shown and compared by Mann Whitney U Test with LM mice (n=8 experiments). * $p<0.05$

Figure S3

Histograms showing relative amounts of *Atg5* transcripts in isolated $CD4^+$ or $CD8^+$ T cells according to the indicated genotypes: C57BL/6 (B6), Littermate (LM), *Atg5*$^{f/f}$ dLck cre mice (n=3 for each genotype), by quantitative RT-PCR. Means obtained with independent experiments are shown. One control mouse is arbitrarily used as reference and set to 1. Bars represent standard deviation. (n=3 per genotype)

Figure S4

Evaluation of purified CD4+ T cell proliferation monitored by CFSE staining after stimulation by anti-CD3 antibody at two doses (1 and 5 µg/mL), a combination of anti-CD3 and anti-CD28 antibodies or 50 ng/mL PMA and 1 µg/mL ionomycin, for 72 hours.

A Representative histograms showing the percentages of CSFE^{low} cells, i.e. cells that have proliferated.

B Histograms showing relative amounts of *Atg5* transcripts in isolated CD4^{+} T cells according to the indicated genotypes: Littermate (LM), *Atg5$^{f/f}$* dLck cre mice (n=3 for each genotype), by quantitative RT-PCR, at the end of the experiment. Means obtained with independent experiments are shown. Values obtained for one control mouse were arbitrarily used as reference and set to 1. Bars represent standard deviation. (n=4 per genotype)

Figure S5

Mice received OVA in CFA i.p. at day 0, and at day 10 OVA in IFA. Mice were sacrificed 8 weeks after the initial injection and spleens were collected. Stainings were performed to quantify TfH cells (TCRb+CD4+PD1+CXCT5+), germinal center B cells (GB B cells B220+CD19+CD95+GL7+) and plasma cells (B220-CD138+) cells. Representative dot plots are shown (top) for each genotype tested (littermate, LM or *Atg5$^{f/f}$* dLck cre) and a summary of the experiment is shown (bottom). Littermate (LM), *Atg5$^{f/f}$* dLck cre mice are shown and compared by Mann Whitney U Test with LM mice (n=5 per genotype). * $p<0.05$

Figure S6

CD4^{+} T cells were isolated from C57BL/6 (B6) littermate (LM) and *Atg5$^{f/f}$* dLck cre mice and stimulated by anti-CD3 and anti-CD28 Abs for 7 days. Lactate was then measured in the collected supernatant by luminometry assaying lactate reductase activity. (n=4 per genotype)

Figure S7

CD4^{+} T cells were isolated from C57BL/6 (B6) littermate (LM) and *Atg5$^{f/f}$* dLck cre mice and stimulated by anti-CD3 and anti-CD28 Abs for 7 days. Cell viability was then assessed by flow cytometry by estimating the proportion of Annexin V-/PI- cells. (n=4 per genotype)

Figure S8

$CD4^+$ T cells were isolated from indicated genotypes and stimulated by anti-CD3 and anti-CD28 antibodies for 7 days in the presence of polarizing cytokines to differentiate cells into Th1, Th2 or Th17 cells.

A Longitudinal study of the ratio between percentages of CM T cells and EM T cells among viable cells, measured by flow cytometry, at indicated days for Th1, Th2 and Th17 cells.

B Results obtained with independent experiments for cell survival at the end of the protocol, at day 42. Each point represents an individual measurement, histograms stand for means and bars represent standard deviation. $Atg5^{f/f}$ dLck cre mice are represented and compared to Mann Whitney U Test. ** $p<0.01$ * $p<0.05$
